# Enhancing Hydrogen Evolution Reaction via Synergistic Interaction between the [Mo_3_S_13_]^2–^ Cluster Co-Catalyst and WSe_2_ Photocathode

**DOI:** 10.1021/acsami.2c14312

**Published:** 2022-11-15

**Authors:** Fanxing Xi, Farabi Bozheyev, Xiaoyu Han, Marin Rusu, Jörg Rappich, Fatwa F. Abdi, Peter Bogdanoff, Nikolas Kaltsoyannis, Sebastian Fiechter

**Affiliations:** † Institute for Solar Fuels, Helmholtz-Zentrum Berlin für Materialien und Energie GmbH, Hahn-Meitner-Platz 1, 14109Berlin, Germany; ‡ Institute of Photoelectrochemistry, Helmholtz-Zentrum Hereon, 21502Geesthacht, Germany; § National Nanolaboratory, al-Farabi Kazakh National University, 71 al-Farabi Ave., 050000Almaty, Kazakhstan; ∥ Department of Chemistry, The University of Manchester, Oxford Road, ManchesterM13 9PL, U.K.; ⊥ Department Structure and Dynamics of Energy Materials, Helmholtz-Zentrum Berlin für Materialien und Energie GmbH, Hahn-Meitner-Platz 1, 14109Berlin, Germany; # Institute Silicon Photovoltaics, Helmholtz-Zentrum Berlin für Materialien und Energie GmbH, Magnusstrasse 12, 12489Berlin, Germany; ∇ PV ComB, Helmholtz-Zentrum Berlin für Materialien und Energie GmbH, Schwarzschildstrasse 3, 12489Berlin, Germany

**Keywords:** molybdenum sulfide, heterojunction, hydrogen evolving catalyst, solar water splitting, photoelectrochemistry

## Abstract

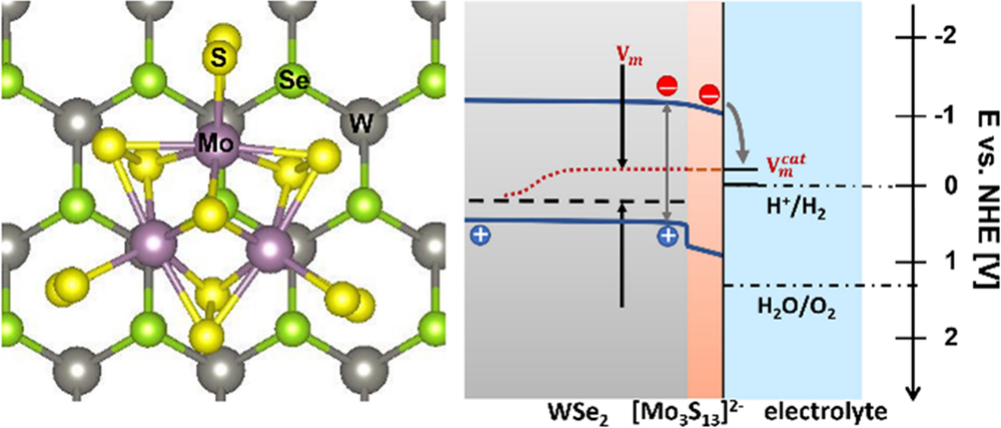

A thiomolybdate [Mo_3_S_13_]^2–^ nanocluster is a promising catalyst for hydrogen evolution reaction (HER) due to the high number of active edge sites. In this work, thiomolybdate cluster films are prepared by spin-coating of a (NH_4_)_2_Mo_3_S_13_ solution both on FTO glass substrates as hydrogen evolving electrodes and on highly 00.1-textured WSe_2_ for photoelectrochemical water splitting. As an electrocatalyst, [Mo_3_S_13_]^2–^ clusters demonstrate a low overpotential of 220 mV at 10 mA cm^–2^ in 0.5 M H_2_SO_4_ electrolyte (pH 0.3) and remain structurally stable during the electrochemical cycling as revealed by in situ Raman spectroscopy. Moreover, as a co-catalyst on WSe_2_, [Mo_3_S_13_]^2–^ clusters enhance the photocurrent substantially by more than two orders of magnitude (from 0.02 to 2.8 mA cm^–2^ at 0 V vs RHE). The synergistic interactions between the photoelectrode and catalyst, i.e., surface passivation and band bending modification by the [Mo_3_S_13_]^2–^ cluster film, promoted HER catalytic activity of [Mo_3_S_13_]^2–^ clusters influenced by the WSe_2_ support, are revealed by intensity-modulated photocurrent spectroscopy and density functional theory calculations, respectively. The band alignment of the WSe_2_/[Mo_3_S_13_]^2–^ heterojunction, which facilitates the electron injection, is determined by correlating UV–vis with photoelectron yield spectroscopy results.

## 1 Introduction

Hydrogen (H_2_) is an essential feedstock for various industries, e.g., petroleum refining, and the synthesis of NH_3_ for fertilizer production.^1,2^ Meanwhile, it is a promising alternative to replace fossil fuels as an energy carrier, due to its high gravimetric energy density and zero carbon dioxide (CO_2_) emission during its combustion. In addition, the urge to decarbonize steel production also creates a gigantic demand for H_2_ as a reducing agent.^3^ However, the majority of H_2_ today (>50%) is produced using steam methane reforming, which consumes unsustainable natural gas and emits CO_2_.^4−6^ H_2_ production methods that are cheap, renewable, and environmentally friendly, e.g., electrochemical (EC) and photoelectrochemical (PEC) water splitting, are therefore desired and have indeed drawn significant research attention.^7−11^

In both EC and PEC methods, suitable catalysts are needed to minimize the kinetic overpotential (η)^12−14^ for hydrogen evolution reaction (HER). Among the many alternatives, including metals (Ru@ MHC, Rh, NiMo, and IrCo), metal sulfides, and phosphides (Co, Fe, Ni, and Mo), molybdenum sulfide stands out due to its high catalytic activity, precious-metal free composition, and good stability in acidic solutions.^15−23^ Tributsch et al.^24,25^ first introduced molybdenum sulfide as a catalyst for HER in 1977. Since then, efforts have been put into elucidating the catalytic mechanism and improving the catalytic activity. In 2007, Jaramillo et al.^26^ proposed that the active sites in MoS_2_ are located on the edges of the hexagonal S–Mo–S layer entities, while the sulfur atoms forming the structural basal planes are catalytically inert. In 2014, a [Mo_3_S_13_]^2–^ nanocluster catalyst was acquired from a (NH_4_)_2_Mo_3_S_13_ precursor, which was first synthesized by Müller et al. in 1978.^27,28^ This nanocluster catalyst consists of a high ratio of active edge sulfur atoms, which was attributed as the reason for its low overpotentials (180–220 mV at a current density of 10 mA cm^–2^, η_10_).^1^ The importance of the [Mo_3_S_13_]^2–^ cluster was further supported in amorphous molybdenum sulfide catalysts. For example, Yeo and co-workers^29^ and Artero and co-workers^30^ reported high activity electrodeposited MoS_*x*_ catalysts with a polymeric structure consisting of [Mo_3_S_13_]^2–^ clusterlike entities. Recently, current authors also reported that amorphous MoS_*x*_ prepared by reactive magnetron sputtering features similar entities of [Mo_3_S_13_]^2–^ cluster material. Despite its high performance as an HER catalyst exhibiting an overpotential of 180 mV at 10 mA cm^–2^ in 0.5 M H_2_SO_4_, the clusterlike structure in an amorphous matrix transforms into MoS_2–*x*_ nanoparticles during the EC process.^31^ Therefore, it forms part of the rationale of the current work to investigate the [Mo_3_S_13_]^2–^ cluster catalyst, prepared via a different approach using a (NH_4_)_2_Mo_3_S_13_ precursor, with respect to its catalytic activity, active site determination, and structural stability.

Furthermore, [Mo_3_S_13_]^2–^ clusters can in addition be used as a co-catalyst on a photoactive cathode. Benck et al.^17^ used [Mo_3_S_13_]^2–^ nanoclusters on top of MoS_2_-silicon photoelectrodes which decreased the photocurrent onset potential by 80 mV. However, the catalytic mechanism of the cluster on a photoactive electrode and the interaction between the cluster material and the photoelectrode remain partially unknown. Therefore, it is the second rationale of this study to investigate the [Mo_3_S_13_]^2–^ nanoclusters as a co-catalyst on a p-type photocathode for PEC water splitting.

Herein, WSe_2_ has been chosen as the photoactive cathode material because of its suitable band gap of 1.5–1.9 eV, a high optical absorption coefficient (∼10^5^ cm^–1^), and a high catalytic ability for hydrogen evolution.^32−34^ [Mo_3_S_13_]^2–^ nanoclusters were then spin-coated on a WSe_2_ photocathode which was previously prepared via an amorphous solid–liquid–crystalline solid (aSLcS) process.^35,36^ After the nanocluster deposition, a photocurrent increase of over two orders of magnitude (from 0.02 to 2.8 mA/cm^2^) at 0 V vs RHE was observed. To understand the underlying mechanisms, we applied various experimental methods, such as intensity-modulated photocurrent spectroscopy (IMPS), photoelectron yield spectroscopy (PYS), and theoretical calculations using density functional theory (DFT). We found that such a dramatic improvement can be attributed to the synergistic interaction between the WSe_2_ support and the thiomolybdate catalyst, whereas the [Mo_3_S_13_]^2–^ clusters passivate surface sites of the WSe_2_ photocathode and improves the light-driven charge separation by modifying the band structure, while WSe_2_ promotes the HER catalytic activity of the [Mo_3_S_13_]^2–^ clusters possibly by modifying the hydrogen adsorption free energy () of both terminal and bridging sulfur atoms as active sites.

## 2 Results and Discussion

### 2.1 Structural Characterization Study of [Mo_3_S_13_]^2–^ Electrodes

**Figure 1:**
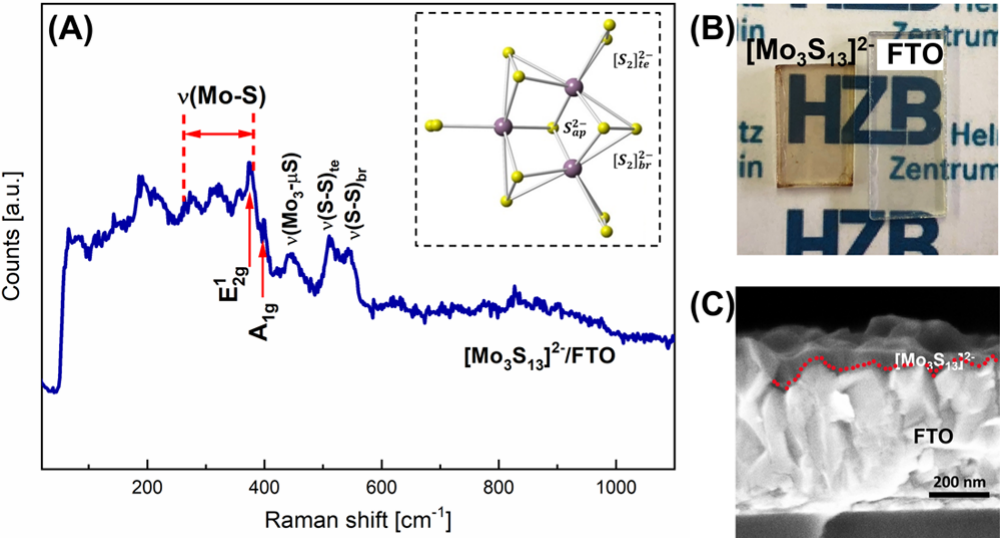
(A) Raman spectrum of the spin-coated [Mo_3_S_13_]^2–^ cluster film on FTO; Inset: Structure of the [Mo_3_S_13_]^2–^ cluster unit (Mo atoms (purple); S atoms (yellow)); (B) photograph of a spin-coated [Mo_3_S_13_]^2–^ layer on FTO (left) in comparison to the bare FTO substrate (right); (C) cross-section SEM image of the spin-coated [Mo_3_S_13_]^2–^ film on the FTO substrate.

A typical [Mo_3_S_13_]^2–^ unit consists of three kinds of sulfur ligands, namely, terminal [S_2_]^2–^ ligand (*n* = 3) that bonds to only one Mo atom, bridging [S_2_]^2–^ ligand (*n* = 3) that connects two Mo atoms, and apical S^2–^ ligand (*n* = 1) that neighbors all three Mo atoms. The validity of the structure can therefore be justified by detecting the characteristic bonds using Raman spectroscopy. As shown in Figure 1A, the vibration modes identified at ∼510, ∼542, and ∼450 cm^–1^ can be assigned to terminal [S_2_]^2–^ (ν(S-S)_te_), bridging [S_2_]^2–^ (ν(S-S)_br_), and bonding between the Mo atom and apical S atom (ν(Mo_3_-μS)),^30^ respectively. In the precursor phase, the thiomolybdate clusters occur as dark red (NH_4_)_2_Mo_3_S_13_ crystal powder (see Figures S1 and S2A,B for image and XRD characterization). When deposited on FTO or on WSe_2_ using spin coating, the crystallinity is lost according to the XRD pattern in Figure S2B, but the cluster structure remains as shown by the characteristic bonds in the Raman spectrum. Figure 1B shows the appearance of a homogeneously spin-coated layer on the FTO glass substrate, which has a thickness of ∼50 nm according to the cross-section scanning electron microscopy (SEM) image in Figure 1C.

### 2.2 Electrocatalytic Property of the [Mo_3_S_13_]^2–^ Cluster Electrode

**Figure 2:**
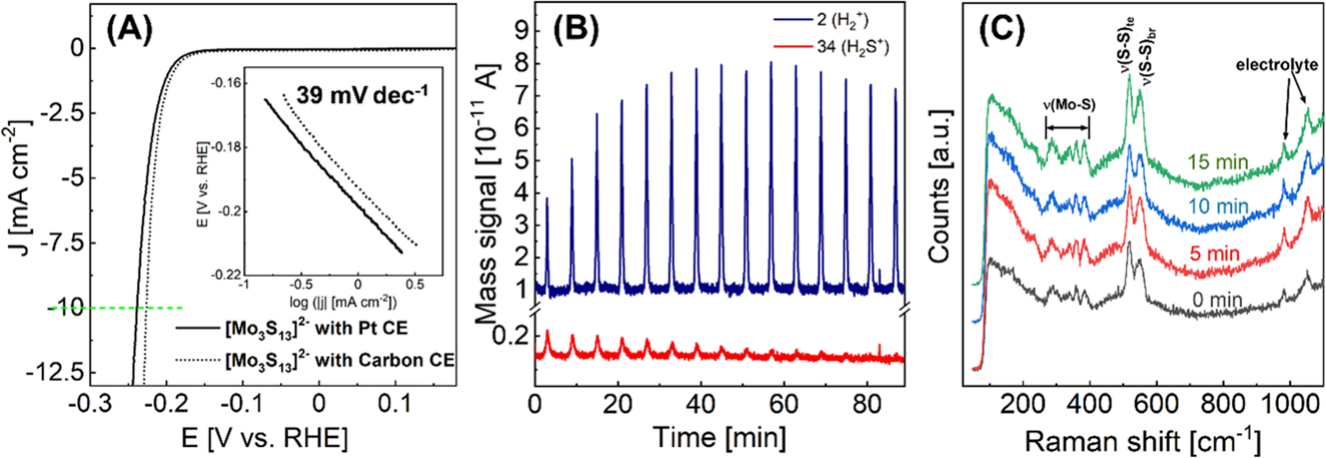
(A) iR-corrected cyclic voltammograms (CV) and Tafel plots derived from the CV curves of [Mo_3_S_13_]^2–^ cluster electrodes prepared using the DMSO solvent via spin-coating; (B) DEMS result of the [Mo_3_S_13_]^2–^ film electrode during 15 cycles of CV measurements. Each CV cycle was performed from 0.2 to −0.2 V vs RHE with a slow scan rate of 2 mV s^–1^ to match the ∼10 s response delay of the mass spectrometer. Note that the small spike appearing at around 83 min is caused by a refreshment of the electrolyte; (C) in situ Raman spectra of a [Mo_3_S_13_]^2–^ cluster film in the beginning and after EC cycling which stand for the unmeasured electrode as well as for the spectra recorded after 5, 10, and 15 min of CV, respectively.

Figure 2A shows the cyclic voltammetry images of the [Mo_3_S_13_]^2–^ catalyst electrode prepared by spin-coating using dimethyl sulfoxide (DMSO) as the solvent. The electrode prepared in this way shows superior activity compared to other methods (see Figure S3). Both Pt and glassy carbon were used as the counter electrode in the tests to rule out the possibility that Pt could be redeposited on the working electrode, thus influencing the catalytic performance during EC cycling.^37^ Nearly identical CV curves were obtained for both tests using Pt and glassy carbon, yielding overpotentials of 235 and 220 mV at −10 mA cm^–2^ (η_10_), respectively, which is in line with the reported range for [Mo_3_S_13_]^2–^ cluster catalysts.^1,38^ We note that the catalytic performances obtained here are still lower than that of Jaramillo and co-workers^1^ (η_10_ = 180 mV), possibly because the flat FTO substrate used in our study has a smaller surface area and lower charge transport properties than the Toray graphite paper used in their study.^1^

The inset of Figure 2A shows the Tafel plots derived from the CV curves using the applied potential (*E*) vs the logarithm of the absolute current density (log |*j*|). The slopes of the Tafel plots can be correlated with the HER mechanisms of the catalyst by elucidating the rate-limiting step in hydrogen evolution (see the detailed discussion in the Supporting Information (SI)).^39,40^ In the current tests of the [Mo_3_S_13_]^2–^ catalyst, a Tafel slope of 39 mV dec^–1^ is obtained, which suggests that the hydrogen evolution follows the Volmer–Heyrovsky mechanism with the Heyrovsky step being the rate-limiting step. Please note that the CV curves and the Tafel slopes of Figure 2A are taken after the CV currents became stable, and the contribution of hydrogen sulfide formation was negligible.

The gaseous products obtained during the CV measurements are further analyzed by differential electrochemical mass spectrometry (DEMS) measurements. Mass/charge ratios 2 and 34 were detected in the DEMS results (Figure 2B), which correspond to H_2_^+^ and H_2_S^+^, respectively. At the beginning of the CV measurements (first cycle), both signals of H_2_ and H_2_S can be observed. In the further cycling of the CV measurements, the production of H_2_ is gradually increased and saturated after about 6 cycles, while the H_2_S generation is steadily reduced.

The formation of H_2_S suggests that part of the sulfur species in the catalyst are released during the CV measurements. To identify which part of the sulfur species reacts with protons and if such a reaction influences the integrity of the cluster unit structure (Figure 1A), in situ Raman spectroscopy was performed before and after EC cycling, with the focus on the change of the vibration modes characteristic to the [Mo_3_S_13_]^2–^ cluster unit, namely, ν(S-S)_te_ and ν(S-S)_br_. As shown in the in situ Raman spectra (Figures 2C and S4), no pronounced change after CV cycling and long-term EC measurements under turnover conditions can be identified on the vibration modes of concern. This indicates that the structure of the spin-coated [Mo_3_S_13_]^2–^ clusters remains stable during EC measurements, in contrast to the sputtered amorphous MoS_*x*_ that almost completely lost its clusterlike structure after EC cycling.^31^

X-ray photoelectron spectroscopy (XPS) was further used to determine the origin of the sulfur atoms contributing to the H_2_S formation. According to the S2p spectra of the catalyst before and after EC cycling (Figure S5), the signals of terminal [S_2_]^2–^, bridging [S_2_]^2–^, and apical S^2–^ entities show little changes, but the signal of the sulfur residue is removed after 10 CV cycles. Therefore, the released sulfur during CV can be interpreted as the sulfur residue from the ammonium polysulfide used for precursor preparation, in accordance with the findings of Kibsgaard et al.^1^ As the sulfur residue is gradually removed after several CV cycles, we speculate that more active sites are exposed, thus enhancing the hydrogen production activity of the catalyst (Figure 2B).

Since the [Mo_3_S_13_]^2–^ cluster structure in our catalyst is maintained during the EC cycling, the electrocatalytic activity of the catalyst is attributed to the terminal sulfur ligands. Indeed, the Gibbs free energy of hydrogen adsorption (, see Table S1) of the terminal sulfur ligands is closest to zero. This implies that hydrogen could be easily adsorbed in the Volmer step and released from the surface as hydrogen gas as shown by previous DFT calculations.^31^

### 2.3 Thiomolybdate Cluster Units as the Co-Catalyst on p-Type WSe_2_ Electrodes

#### 2.3.1 Enhanced HER Performance of [Mo_3_S_13_]^2–^-Cluster-Modified WSe_2_

**Figure 3:**
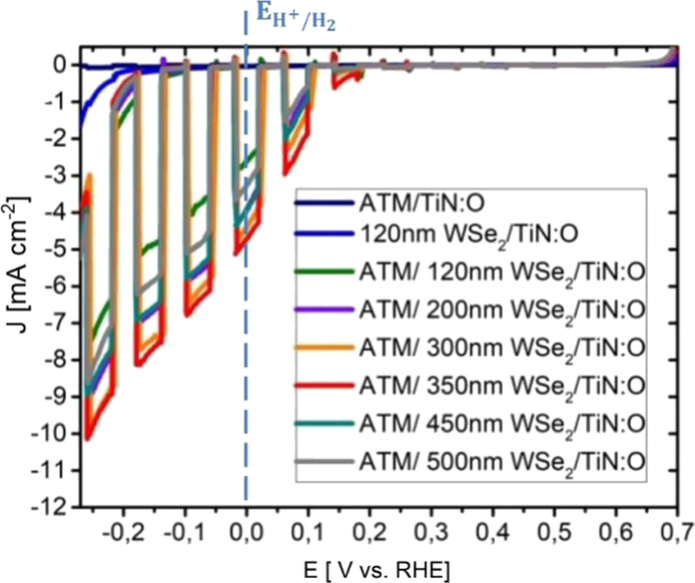
Linear sweep voltammetry (LSV) of bare [Mo_3_S_13_]^2–^ (dark blue curve) and bare WSe_2_ (blue curve) and WSe_2_/[Mo_3_S_13_]^2–^ photoelectrodes (green to gray curves) under front-side illumination conditions using chopped light (AM 1.5) and a 0.5 M H_2_SO_4_ (pH = 0.3) aqueous electrolyte (reproduced with permission.^41^ 2019, Royal Society of Chemistry).

The PEC performance of the WSe_2_ electrode can be vastly improved by depositing the thiomolybdate [Mo_3_S_13_]^2–^ cluster catalyst, as revealed by linear sweep voltammetry (LSV) measurements in our previous work.^41^ The results are adapted and presented here in Figure 3. For the bare WSe_2_ electrode (120 nm), the photocurrent is very small (∼0.02 mA cm^–2^ at 0 V vs RHE, see the magnified LSV curve of bare WSe_2_ in Figure S6). The dark current starts at about −0.16 V vs RHE. Similar behavior has been demonstrated in another report.^42^ Such low photocurrent of WSe_2_ can be attributed to inert van der Waals planes exposed and high recombination rates, possibly resulting from the formation of PdSe_*x*_ during the sputtering process by the reaction gas (H_2_Se) and Pd which serves as the promoter for WSe_2_ crystallization.^43^ After depositing [Mo_3_S_13_]^2–^ clusters on the electrode, the photocurrent measured at 0 V vs RHE increases to ∼2.8 mA cm^–2^ (WSe_2_ of 120 nm, green curve), which is more than 2 orders of magnitude higher than that of bare WSe_2_. A further improvement to 5.6 mA cm^–2^ (at 0 V vs RHE) can be obtained by optimizing the thickness of the WSe_2_ film electrode (350 nm, red curve). This optimized thickness is explained by a trade-off between the charge carrier diffusion length of ∼400 nm for electrons and holes and the light absorption in the WSe_2_ film defining the number of excited electron–hole pairs.^40^ We attribute such significant PEC performance enhancement of the WSe_2_ after [Mo_3_S_13_]^2–^ deposition to a synergy between the [Mo_3_S_13_]^2–^ catalyst film and the WSe_2_ electrode, which will be discussed in detail in the following sections, while the detailed fabrication process and optical and PEC characterization results of WSe_2_ and WSe_2_/[Mo_3_S_13_]^2–^ can be found in the previous publication and in the SI (Figures S7–S11).^41^

#### 2.3.2 Mechanistic Insights into the Enhanced PEC Performance of the WSe_2_/Thiomolybdate Photoelectrode

In general, the functions of a co-catalyst are as follows: (i) improving the charge transfer of photogenerated charge carriers from the photoactive absorber layer to the surface of the electrode; (ii) suppressing surface recombination of excited charge carriers at the semiconductor/electrolyte interface; and (iii) generating a photoelectrode/catalyst heterojunction leading to an advantageous band bending.

Consequently, the deposition of the thiomolybdate catalyst could either improve the charge separation efficiency η_CS_, namely, the fraction of photogenerated minority charges that reach the electrode surface, or the charge transfer efficiency η_CT_ which is defined as the fraction of minority charges transferred from the semiconductor surface into the electrolyte. The value of η_CS_ is normally dependent on the diffusion length of the minority charge carriers in the semiconductor and the width of the space charge region.^44^ The η_CT_ value can be determined by the charge transfer rate (*k*_tr_) and the surface recombination rate (*k*_rec_) at the electrode/electrolyte interface:
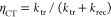
1

**Figure 4:**
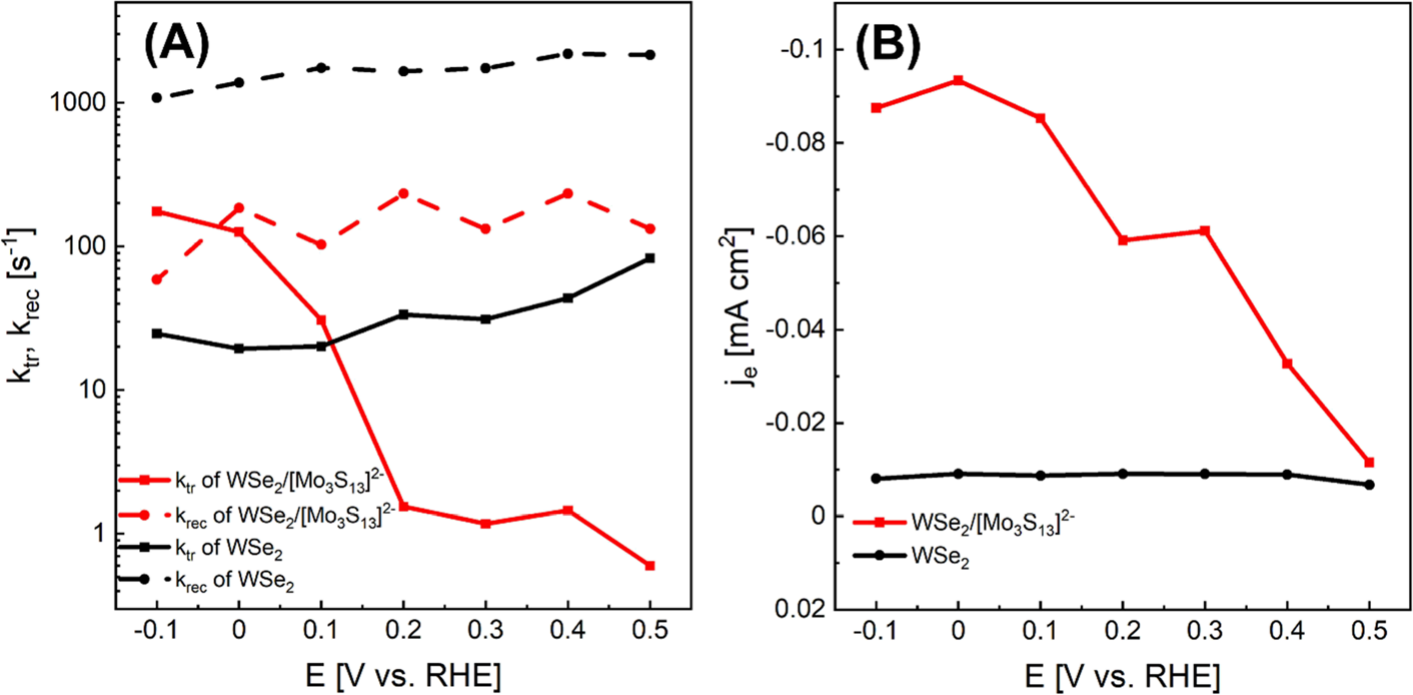
(A) Calculated charge transfer rates (*k*_tr_, solid curves) and surface recombination rates (*k*_rec_, dashed curves) as a function of applied potential deviated from IMPS spectra of WSe_2_ (black curve) and WSe_2_/[Mo_3_S_13_]^2–^ (red curve); (B) electron current densities (*j*_e_) of the WSe_2_ (black curve) and the WSe_2_/[Mo_3_S_13_]^2–^ (red curve) electrode inferred from IMPS spectra.

To study the evolutions of the charge transfer rate (*k*_tr_) and the surface recombination rate (*k*_rec_) as a function of the applied potential, we performed IMPS^45^ measurements. Since HER is not a single electron transfer reaction, the values of *k*_tr_ and *k*_rec_ were obtained using a “phenomenological approach” as a simplified model which has been proven useful in various PEC water splitting studies, for example, for p-Si, p-InP, BiVO_4_, Fe_2_O_3_, and ZnFe_2_O_4_.^44−49^ In this approach, the *k*_tr_ and *k*_rec_ values were treated as pseudo first-order rate constants which are functions of the real rate constants of the elementary steps.^50,51^ The results for both the bare WSe_2_ photocathode and WSe_2_ with a [Mo_3_S_13_]^2–^ catalyst film are shown in Figure 4A. The raw IMPS spectra and the method to determine these parameters are documented in the Supplementary Information (Figure S12, eq S4).

In line with the previous work,^41^ our IMPS results here also show a very high recombination rate for the bare WSe_2_ electrode (Figure 4A). After the deposition of the [Mo_3_S_13_]^2–^ catalyst, the recombination rate of the photocathode is decreased by about a factor of 10 in the entire potential range (0.5 to −0.1 V vs RHE). Meanwhile, the influence of the [Mo_3_S_13_]^2–^ cluster on *k*_tr_ is more complex. At a positive potential ranging from 0.2 to 0.5 V vs RHE, the *k*_tr_ of the WSe_2_/[Mo_3_S_13_]^2–^ photocathode is significantly lower than the one of bare WSe_2_. However, as the potential goes to more cathodic values, the *k*_tr_ of the WSe_2_/[Mo_3_S_13_]^2–^ photocathode starts to increase rapidly and becomes higher than that of the bare WSe_2_ electrode at a potential <0.1 V vs RHE. At −0.1 V vs RHE, *k*_tr_ increases by one order of magnitude. The exact reason for the change of *k*_tr_ is not yet clarified, but overall, the influence of depositing the [Mo_3_S_13_]^2–^ cluster co-catalyst on the WSe_2_ photocathode to the rate constants corresponds well with the significant improvement of the photocurrent density shown in Figure 3.

In addition to the change of *k*_tr_ and *k*_rec_, the electron current density (*j*_e_) of the WSe_2_/[Mo_3_S_13_]^2–^ electrode is also larger than that of the bare WSe_2_ photocathode. *j*_e_ can be obtained from the absolute intercepts of the high frequency semicircles with the *x* axis in IMPS; this represents the flux of electrons that arrive at the semiconductor interface before they can recombine (or transfer to the electrolyte; see the example in Figure S13). In Figure 4B, the *j*_e_ values from both types of electrodes are plotted versus the applied potentials. At 0.5 V vs RHE, the electron current densities of these two photoelectrodes are similar. However, when the potential moves in the cathodic direction, and *j*_e_ of WSe_2_ remains almost constant and below 0.01 mA cm^–2^ (black curve), while *j*_e_ of the WSe_2_/[Mo_3_S_13_]^2–^ electrode (red curve) increases continuously and reaches a value of about 0.1 mA cm^–2^ at a potential of 0 V vs RHE. If the [Mo_3_S_13_]^2–^ layer only passivates surface states of the WSe_2_ photocathode or increases the charge transfer rate at the interface, a similar *j*_e_ should be observed in both samples, given that the light absorption of the WSe_2_ photocathodes stays similar with or without the thin catalyst layer. We therefore attribute the significant difference in Figure 4B to the increase of the charge separation efficiency (η_CS_) caused by the change of band bending in the electronic structure of the photocathode when covered by the catalyst layer.

To unveil the change of the band bending after the deposition of [Mo_3_S_13_]^2–^ on WSe_2_, the open-circuit potential difference (ΔOCP) between illumination and dark conditions was measured for both samples. A higher ΔOCP is indeed observed for the WSe_2_/[Mo_3_S_13_]^2–^ electrode than bare WSe_2_ (∼150 vs ∼20 mV, see Figure S14 for raw data), thus verifying the change of the band bending caused by the formation of the WSe_2_/[Mo_3_S_13_]^2–^ heterojunction.

**Figure 5:**
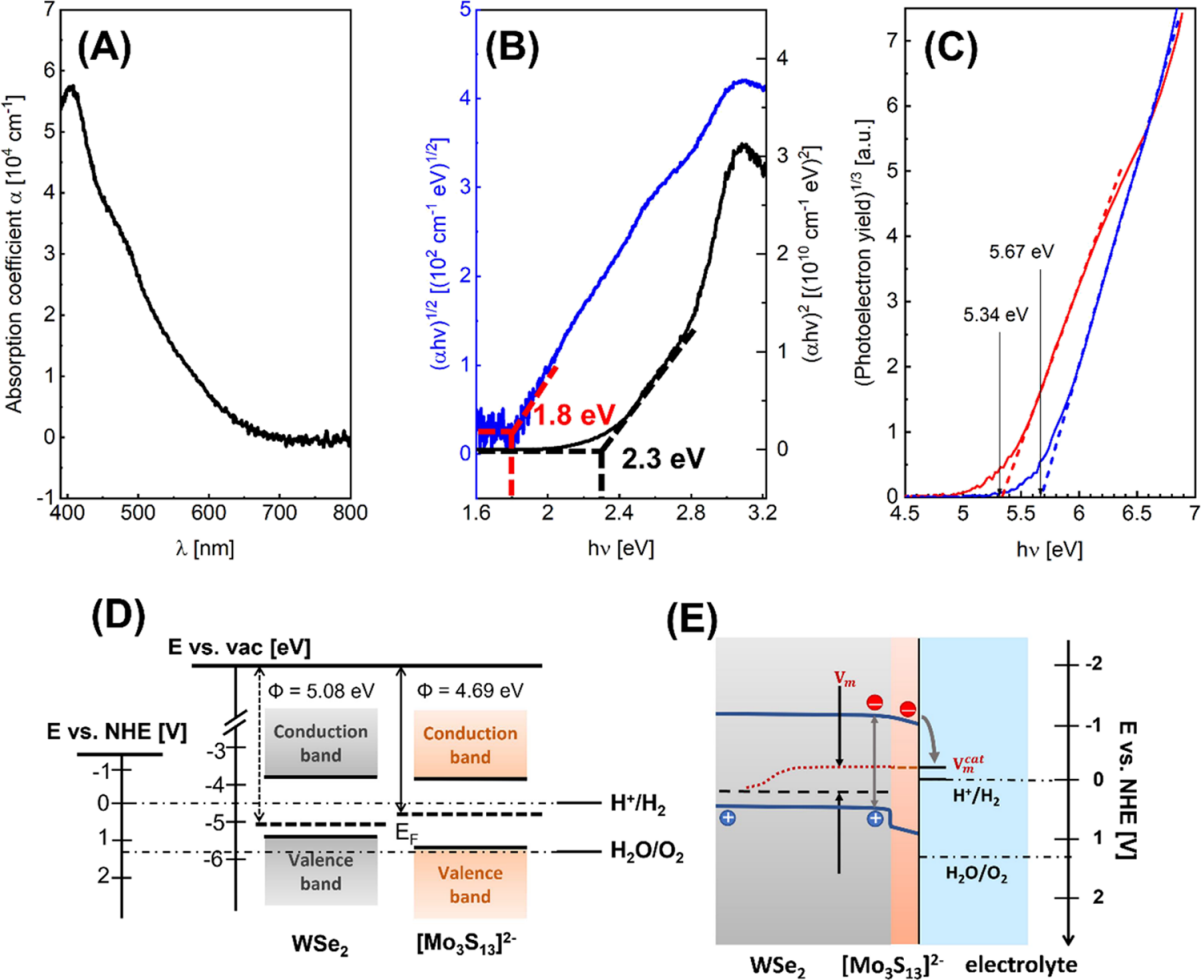
(A) Absorption coefficient (α) plotted as a function of wavelength (λ) of the [Mo_3_S_13_]^2–^ cluster film; (B) Tauc plot of the [Mo_3_S_13_]^2–^ film indicating an indirect and direct optical transition deviated from the slopes of the curves; (C) photoelectron yield spectra (PYS) of WSe_2_ (red) and [Mo_3_S_13_]^2–^ (blue) presented with the cubic root of the photoelectron yield as a function of photon energy (*h*ν); (D) energy level diagram of the intrinsic [Mo_3_S_13_]^2–^ cluster film and the p-type WSe_2_ absorber film with respect to the vacuum level and normal hydrogen electrode (NHE); (E) scheme of a band alignment in the WSe_2_/[Mo_3_S_13_]^2–^ heterojunction in acidic electrolyte under illumination. The red dotted line indicates the course of a quasi-Fermi level.

The band positions of the WSe_2_ layer and the [Mo_3_S_13_]^2–^ cluster film have been investigated separately to determine the band alignment at the WSe_2_/[Mo_3_S_13_]^2–^ interface. The band gap of [Mo_3_S_13_]^2–^ was inferred from UV–vis absorption measurements. Figure 5A,B shows the absorption coefficient and the Tauc plot acquired from the UV–vis measurement of the 50 nm thick cluster film on the FTO substrate. The [Mo_3_S_13_]^2–^ cluster layer starts to absorb light from 700 nm, and its absorption coefficient peaks at a wavelength of 410 nm, reaching ∼5.5 × 10^4^ cm^–1^. As derived from the Tauc plot (Figure 5B), the [Mo_3_S_13_]^2–^ catalyst thin film shows an indirect band gap of 1.8 eV and a direct one of 2.3 eV. The valence band maximum (VBM) relative to the local vacuum level, i.e., the ionization energy (IE), was determined from PYS.^52^ The local vacuum level was defined by the work function (Φ) of the materials extracted from Kelvin Probe (KP) measurements.^53^ The VBM position relative to the Fermi level can then be calculated from the difference Φ-IE. With the known VBM position, the conduction band minimum (CBM) was obtained by adding the band gap of the materials to the previously determined VBM value. According to the PYS results shown in Figure 5C, the VBM of the [Mo_3_S_13_]^2–^ catalyst film is located at 5.67 eV vs the local vacuum level. The work function of the catalyst was measured to be 4.69 ± 0.03 eV by the contact potential difference (CPD) method using a Kelvin probe, which is shown in Figure S15. Combining all these results (UV–vis, CPD, and PYS measurements), the band positions of the [Mo_3_S_13_]^2–^ catalyst film could be constructed as shown in Figure 5D. The catalyst film shows an intrinsic semiconducting behavior since the Fermi level lies close to the mid gap position. Figure 5D also shows the band positions for WSe_2_ using the VBM value (5.34 ± 0.03 eV) obtained from the PYS measurement (Figure 5C, red curve), its work function (5.08 ± 0.04 eV) determined from the CPD measurement (Figure S16) which is ∼0.4 eV higher than the work function of the [Mo_3_S_13_]^2–^ catalyst film, and the band gap (∼1.5 eV^41^).

With the positions of the [Mo_3_S_13_]^2–^ clusters and WSe_2_, a band diagram of the WSe_2_/[Mo_3_S_13_]^2–^ heterojunction under illumination can be constructed as shown in Figure 5E. The band bending in WSe_2_ is small compared to that in [Mo_3_S_13_]^2–^ owing to the orders of magnitude difference of the doping concentrations in the materials. Being an intrinsic semiconductor, a thin layer of [Mo_3_S_13_]^2–^ cluster film deposited on WSe_2_ is therefore completely depleted. The conduction and valence band edges in the [Mo_3_S_13_]^2–^ film turn downward which facilitates the electron injection into the electrolyte where protons are reduced to H_2_. At the same time, the rather positive position of the valence band of the [Mo_3_S_13_]^2–^ cluster prevents photogenerated holes to migrate to the catalyst/electrolyte interface, and consequently, the surface recombination at the WSe_2_/[Mo_3_S_13_]^2–^ interface is essentially reduced.

#### 2.3.3 WSe_2_-Influenced Higher Catalytic Activity of the [Mo_3_S_13_]^2–^ Cluster

In order to investigate the performance enhancement at the atomic level, the hydrogen adsorption free energy on various S sites of the thiomolybdate cluster influenced by the interaction between the cluster unit and WSe_2_ monolayer support has been simulated (Figure S17). Previously in the literature, the influence of electrode supports on the catalytic activity was studied by Hellstern et al.^54^ The [Mo_3_S_13_]^2–^ catalyst deposited on an Au substrate obtained the highest HER activity compared to [Mo_3_S_13_]^2–^ on Ag, C, and Cu supports due to a modified H adsorption free energy ().

**Figure 6:**
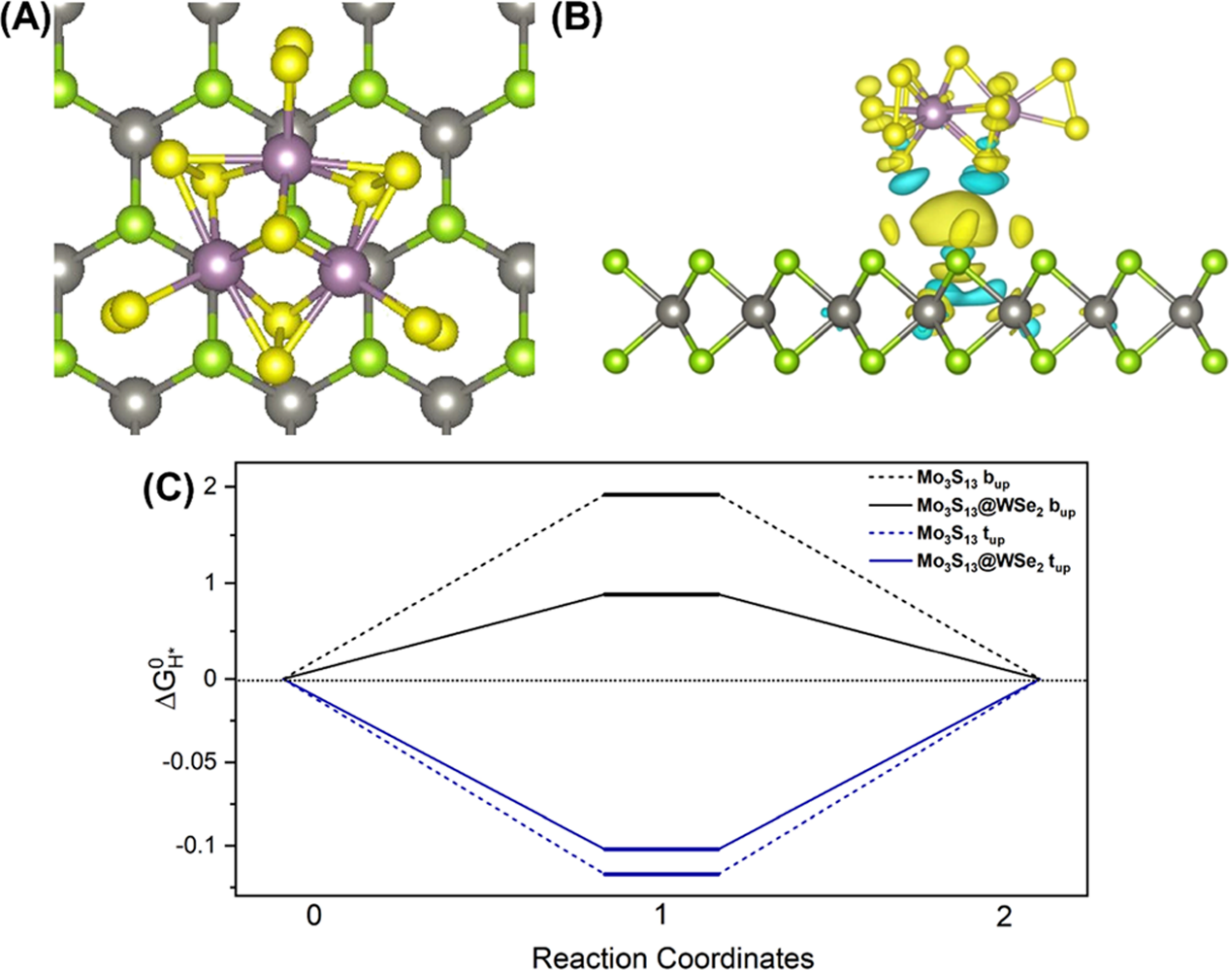
(A) Most stable site of a Mo_3_S_13_ cluster unit on top of a 00.1-oriented WSe_2_ surface as obtained from DFT calculations. The green, gray, yellow, and purple balls represent the Se, W, S, and Mo atoms, respectively. (B) Charge difference of the most stable case in Figure 6A. The yellow and blue areas represent the charge accumulation and decrease, respectively. The isosurface is set to 0.002e/*R*_0,_^3^ where *R*_0_ is the Bohr radius. (C)  on terminal (blue) and bridging (black) upside sulfur atoms on Mo_3_S_13_ (dotted line) and Mo_3_S_13_@WSe_2_ (solid line) from DFT calculations.

In the first step, six possible adsorption sites of the cluster unit based on the 00.1-oriented van der Waals surface of WSe_2_ have been considered as illustrated in Figure S17. The adsorption energy was calculated based on the equation:
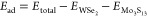
2where *E*_total_, *E*_WSe_2__,and *E*_Mo_3_S_13__ are the energies of the combined system, WSe_2_ monolayer, and Mo_3_S_13_ cluster, respectively. The results are listed in Table S2. After relaxations, all possible sites in Figure S18 are energetically favorable for hosting the cluster with adsorption energies less than 1 eV, especially the combination in Figure 6A where the apical S of [Mo_3_S_13_]^2–^ facing out of plane and located on top of a Se atom of the Se–W–Se unit shows the lowest adsorption energy indicating the most stable case. In the case, the terminal and bridging S atoms are allocated in the middle on top of the trigonal selenium rings of the van der Waals plane.

After loading the catalyst on WSe_2_, the charge transfer between the molecule and the substrate in the most stable case has been investigated by Bader charge analysis and charge density differences.^55^ As shown in Table S3, charges of all the Mo and S atoms close to the WSe_2_ substrate remain unchanged. On the other hand, the charges of the up bridging and terminal S atoms, which are exposed for reaction, increase. Further charge density difference analysis also confirms the charge transfer from the WSe_2_ substrate to Mo_3_S_13_ (Figure 6B).

Thus, the hydrogen adsorption free energy for the most stable case in the Volmer step (eq S1) after the charge transfer was calculated based on
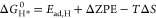
3where *E*_ad, H_ is the hydrogen adsorption energy on different active sites of the Mo_3_S_13_ cluster on WSe_2_, and ΔZPE and Δ*S* are the changes of the zero-point energy and entropy, respectively. The temperature is set to 300 K. The results are listed in Table S1. According to the Sabatier principle,  should be close to zero to achieve high HER activity. As shown by the blue lines in Figure 6C, compared with free-standing Mo_3_S_13_, the most active sites in the cluster on top of WSe_2_ are still the terminal S atoms but with a free energy 15 mV closer to zero (see detailed values in Table S1), suggesting a higher HER catalytic activity. The negative value of  demonstrates that the HER reaction is determined by the second step which breaks the H* bond formed in the Volmer step and releasing H_2_. Furthermore, the free energy of the hydrogen adsorption on bridging S atoms dramatically reduces to 0.885 eV as shown by the black line in Figure 6C, which is similar to the Mo site of the MoS_2_ edges, suggesting that the bridging sulfur atoms could also act as potential reactive sites for HER. In such a case, the protonation of the catalyst (Volmer step) would be the key reaction step in HER because of the positive  of the bridging sulfur atoms when acting as reactive sites.^56^ Overall, the DFT calculations reveal the impact of the WSe_2_ support which leads to a higher HER catalytic activity per site on the terminal S atoms and more active sites due to the charge transfer from WSe_2_ to the cluster catalyst. This conclusion is supported by the dark current behavior in LSV curves from Figure S19. By comparing the black curve ([Mo_3_S_13_]^2–^ on TiN/O), the blue curve (WSe_2_ on TiN/O), and the green curve (120 nm WSe_2_/[Mo_3_S_13_]^2–^ on TiN/O), the highest dark current in this negative potential range is found for the green curve due to a higher HER activity.

## 3 Conclusions

In this work, thiomolybdate [Mo_3_S_13_]^2–^ clusters were studied both as an electrocatalyst for HER and as a semiconducting co-catalyst film deposited on a photoactive WSe_2_ electrode to study PEC water splitting. When used as an electrocatalyst, the thiomolybdate cluster material spin-coated on the FTO substrate exhibited an overpotential of 220 mV at 10 mA cm^–2^ in 0.5 M H_2_SO_4_ (pH 0.3). The [Mo_3_S_13_]^2–^ clusters prepared from the (NH_4_)_2_Mo_3_S_13_ precursor are found to be structurally more stable than those prepared by reactive sputtering, as verified by in situ Raman spectroscopy during EC cycling, where little change is observed on the characteristic bridging and terminal [S_2_]^2–^ bonds of the [Mo_3_S_13_]^2–^ cluster entity. When used as a co-catalyst deposited on the WSe_2_ photocathode, the overall PEC performance at 0 V vs RHE is boosted by at least two orders of magnitude compared to a bare WSe_2_ electrode. This substantial enhancement of performance can be attributed to the synergy between the [Mo_3_S_13_]^2–^ co-catalyst and WSe_2_ photocathode. The clusters act not only as a passivation layer which reduces surface recombination of excited charge carriers on the surface of the WSe_2_ electrode but also appear as an intrinsic semiconductor that forms a heterojunction with WSe_2_ and promote electron transport to the electrode/electrolyte interface where hydrogen evolution takes place according to IMPS results. The band alignment of the heterojunction was determined from UV–vis, KP, and PYS measurements. Besides, the [Mo_3_S_13_]^2–^ clusters deposited on WSe_2_ are also more catalytically active than the free-standing clusters, as revealed by DFT calculations: the hydrogen adsorption free energies () on both terminal and bridging S sites are close to 0, thus more favorable in catalyzing hydrogen evolution. Since the synergy between the catalyst and the photoelectrode discovered in this study originates from their interactions, it is therefore considered a general mechanism that can help rationalize the design of other solar-driven water splitting systems with improved properties.

## 4 Experimental Section

### 4.1 Preparation of (NH_4_)_2_Mo_3_S_13_ and WSe_2_ Electrodes

(NH_4_)_2_Mo_3_S_13_ was synthesized based on the description of Müller et al.^27^ modified by Redeker.^57^ In a 250 mL round flask, 4 g of (NH_4_)_6_Mo_7_O_24_·4H_2_O was dissolved in 20 mL of deionized water. Then, 120 mL of ammonium polysulfide (8%, Fisher scientific) was added, and the mixture was heated at 90 °C under reflux overnight. A dark-red precipitate of (NH_4_)_2_Mo_3_S_13_ was obtained and later filtered and washed with ethanol and water. Subsequently, the powder was heated at 80 °C in toluene (50 mL) three times for about 2 h. After filtering and drying, a dark red thiomolybdate powder was obtained. To prepare thin films of the material, the synthesized powder was dissolved in DMSO or MeOH and deposited by spin-coating or drop-casting on FTO substrates and WSe_2_ electrodes.

WSe_2_ electrodes were prepared by the “aSLcS” process using reactive magnetron sputtering with H_2_Se as the reactive gas and silicon wafers as substrates coated with a TiN/O conductive layer. Further synthesis details can be found in Bozheyev et al.^35^

### 4.2 Structural and Electronic Characterization

Field emission scanning electron microscopy was used to measure the top view and cross-section morphology of the electrodes using a LEO GEMINI 1530 instrument from ZEISS employing an acceleration voltage of 5 kV. A Raman system from Horiba (XploRA; light source λ = 532 nm, light intensity 0.112 mW) was used to measure Raman spectra of the samples, and a DILOR LabRAM micro Raman system for in situ measurements (λ = 632.8 nm, light intensity 4.3 or 47.1 mW mm^–2^)) was employed. UV–vis absorption spectra were measured in an integrating sphere using a PerkinElmer Lambda 950 spectrometer. KP and PYS equipment from KP Technology Ltd. was used to determine the work functions and the VBM of the WSe_2_ and [Mo_3_S_13_]^2–^ films, respectively. The KP system employs a 2.0 mm diameter tip with a gold alloy coating which is calibrated on a gold reference sample. The KP is placed in a Faraday cage which screens the external electrical fields. The CPD between the tip and reference sample is measured with a resolution of 1–3 mV. The PYS setup uses the same KP system to detect the photoemission currents as a function of incident photon energy. The light source comprises a deuterium (D2) lamp coupled with a grating monochromator. The range of the incident photon energy is 3.6–6.9 eV, which corresponds to wavelengths ranging from 340 to 180 nm. The photoemission threshold is determined with a resolution of 30–50 meV.

### 4.3 Electrochemical Measurements

A VersaSTAT potentiostat was used for EC measurements. The iR correction for the CV curves was complemented by using ohmic resistance measured from electrochemical impedance spectroscopy at 100 KHz with a voltage perturbation of 10 mV at OCP. PEC measurements were performed with a potentiostat from EG&G (PAR 273A) under AM1.5 illumination provided by a solar simulator from WACOM (type WXS-50S-5H, class AAA). To study the gaseous products during EC measurements, DEMS consisting of a three-electrode EC cell and a differentially pumped vacuum system attached to a mass spectrometer (QMG 220 M1, PrismaPlus 1–100 amu) connected with a porous hydrophobic membrane was used. Further details about the DEMS system can be found in a previous paper.^58^ For ΔOCP measurements, an Ar-ion laser with a wavelength of 457.9 nm was used to provide a high illumination energy with high power density (216 mW/cm^2^) to flatten the band. A light source (Thorlabs M455L3) in the IMPS setup has an intensity of 20 mW cm^–2^ with a wavelength (λ) of 455 nm and a 10% modulated rms amplitude. A beam splitter was used to split the light into two parts: one directed to a highspeed Si photodiode (Thorlabs PDA10A-EC) and the other one passing the PEC cell. A frequency response analyzer (FRA, Solartron 1250, Schlumberger) was used to modulate the light intensity sinusoidally and detect the modulated response. During EC measurements, a Solartron 1286 potentiostat was used to apply potential to the photoelectrode. Then, the photocurrent (*I*_photo_) measured by the potentiostat was recorded by channel 1 of the FRA, while the voltage signal of the high-speed Si photodiode was recorded by channel 2 of the FRA. The opto-electrical gain was derived by dividing the signals from channel 1 and channel 2. Thus, the Iphoto (electron current) can be determined by multiplying the IMPS output by a conversion factor (0.015 V cm^2^ mA^–1^), which was determined by measuring the absolute light intensity using a calibrated photodiode (PD300UV + Ophir Nova II) and the voltage of the high-speed Si photodiode. The theory and analysis details of IMPS were first described by Peter et al.^51,59^

All the EC measurements were performed in a three-electrode configuration with an Ag/AgCl electrode in saturated KCl solution as a reference electrode. Pt and glassy carbon rods were used both as the counter electrode. In addition, 0.5 M sulfuric acid aqueous solution (pH 0.3) was used as the electrolyte. The contacting area of the sample with the electrolyte was fixed at 0.238 cm^2^ by a 0.55 cm diameter O-ring.

### 4.4 Theoretical Calculations

We performed the DFT calculations of the Mo_3_S_13_ clusters on a trigonal 00.1-oriented monolayer of WSe_2_, using the Vienna ab initio Simulation Package.^60^ The projector augmented wave potentials were adopted to describe the ion–electron interaction.^61^ We used the PBE XC functional together with Grimme’s D2 dispersion correction in all calculations.^62,63^ The energy cut-off was set to 500 eV, and the Hellmann–Feynman forces are less than 0.01 eV Å^–1^. The dipole moment correction was set along the WSe_2_ surface. A 15 Å vacuum slab was added to prevent the interlayer interaction in the periodic condition. For the WSe_2_ monolayer, a 7 × 7 supercell was selected to represent the substrate (Figure S12A). The Mo_3_S_13_ molecule possesses two orientations (Figure S12B,C). We denoted the axial S of Mo_3_S_13_ facing outward from the substrate plane as the up position while the axial S facing inward as the down position.

## Supporting Information Available

Further information about XRD, XPS, LSV, CV and Tafel plots, IPCE, UV-vis, gas formation, stability test, IMPS, ΔOCP, Kelvin Probe measurements, and DFT calculations (PDF)
