# Supplementary material for: Enhancing Hydrogen Evolution Reaction via Synergistic Interaction between the [Mo3S13]2– Cluster Co-Catalyst and WSe2 Photocathode
Source: ACS Appl Mater Interfaces. 2022 Nov 15;14(47):52815–24. doi: 10.1021/acsami.2c14312 (PMC9716521; doi:10.1021/acsami.2c14312)
Supplement: Supplementary file 1 — am2c14312_si_001.pdf [file am2c14312_si_001.pdf]

Supporting Information

Enhancing Hydrogen Evolution Reaction via Synergistic Interaction Between $[Mo_3S_{13}]^{2-}$ Cluster Co-catalyst and WSe_2 Photocathode

Fanxing Xi,* Farabi Bozheyev, Xiaoyu Han, Marin Rusu, Jörg Rappich, Fatwa F. Abdi, Peter Bogdanoff, Nikolas Kaltsoyannis, Sebastian Fiechter,*

Fanxing Xi,[,a,g] Farabi Bozheyev,[a,b,c] Xiaoyu Han,[d] Marin Rusu,[e] Jörg Rappich,[f] Fatwa F. Abdi,[a] Peter Bogdanoff,[a] Nikolas Kaltsoyannis,[d] Sebastian Fiechter,[*,a]*

a. Institute for Solar Fuels, Helmholtz-Zentrum Berlin für Materialien und Energie GmbH, Hahn-Meitner-Platz 1, 14109 Berlin.

b. Institute of Photoelectrochemistry, Helmholtz-Zentrum Hereon, 21502 Geesthacht, Germany.

c. National Nanolaboratory, al-Farabi Kazakh National University, 71 al-Farabi Ave., 050000 Almaty, Kazakhstan

d. Department of Chemistry, The University of Manchester, Oxford Road, Manchester, M13 9PL, United Kingdom.

e. Department Structure and Dynamics of Energy Materials, Helmholtz-Zentrum Berlin für Materialien und Energie GmbH, Hahn-Meitner-Platz 1, 14109 Berlin.

f. Institute Silicon Photovoltaics, Magnusstrasse 12, Helmholtz-Zentrum Berlin für Materialien und Energie GmbH, 12489 Berlin.

g. PV ComB, Helmholtz-Zentrum Berlin für Materialien und Energie GmbH, Schwarzschildstrasse 3, 12489 Berlin.

Corresponding author: fanxing.xi@helmholtz-berlin.de; fiechter@helmholtz-berlin.de

Keywords: molybdenum sulfide, heterojunction, hydrogen evolving catalyst, solar water splitting, photoelectrochemistry

Contents

Figure S1. Photograph of synthesized dark red powder of $(NH_4)_2Mo_3S_{13}$ 4

Figure S2. XRD patterns of (A) $(NH_4)_2Mo_3S_{13}$ precursor powder; (B) bare FTO substrate (black); $[Mo_3S_{13}]^{2-}$ cluster film spin-coated on FTO substrate (red). 4

Figure S3. (A) iR-corrected cyclic voltammograms (CV); (B) Tafel plots derived from the CV curves of $[Mo_3S_{13}]^{2-}$ cluster electrodes prepared using DMSO and MeOH as solvents via drop-casting and spin-coating. 5

Figure S4. (a) Current density of $[Mo_3S_{13}]^{2-}$ cluster catalyst on FTO measured in Raman cell under various potential: -0.2 V vs. RHE (red), -0.25 V vs. RHE (blue), -0.3 V vs. RHE (green), -0.2 V vs. RHE (purple); (b) corresponding in-situ Raman spectra measured in the cell with electrolyte (pH 0.3, 0.5 M H_2SO_4) 6

Figure S5. XPS S2p spectra of the $[Mo_3S_{13}]^{2-}$ cluster catalyst on FTO: (A) electrode as-prepared; (B) after 10 CV cycles. Terminal $[S_2]^{2-}$ units are represented by the green curve while bridging $[S_2]^{2-}$ and apical S^{2-} entities are shown by purple curve in the deconvoluted XPS spectra. Residual sulfur is shown by the dark yellow curve in Figure (A). 7

Figure S6. Magnified LSV of a bare WSe_2 photocathode on TiO:N/Si substrate under front side AM1.5 chopped illumination in 0.5 M H_2SO_4 aqueous electrolyte (pH 0.3). 8

Figure S7. Absorptance spectra, IPCE and APCE spectra of WSe_2, $WSe_2/[Mo_3S_{13}]^{2-}$ photocathodes.[2] . 8

Figure S8. (a) Gas formation at the electrode surface; (b) mass signal of the gas products vs. time (s).[2] 9

Figure S9. Stability test of a WSe_2 (200 nm)/$[Mo_3S_{13}]^{2-}$ photocathode at 0 V vs. RHE in 0.5 M H_2SO_4 under AM 1.5 illumination.[2] 9

Figure S10. X-ray photon spectra (XPS) of WSe_2, $WSe_2/[Mo_3S_{13}]^{2-}$ before and after PEC: (a) W 4f, (b) Se 3d, (c) Mo 3d, and (d) S 2p.[2] 10

Figure S11. Ultraviolet photoelectron spectroscopy (UPS) of WSe_2 and $WSe_2/[Mo_3S_{13}]^{2-}$ to determine the valence band edge position.[2] 10

Figure S12. IMPS spectra of (A) a bare WSe_2 and (B) of a $WSe_2/[Mo_3S_{13}]^{2-}$ photocathode under modulated illumination at different potentials vs. RHE. 11

Figure S13. An example of a raw IMPS spectrum of the $WSe_2/[Mo_3S_{13}]^{2-}$ electrode indicating the values of the electron current (j_e) and of ω_{max} by the red dots (also see Eq. 1 and Eq. S4). 13

Figure S14. The open circuit potential difference (ΔOCP) measurements of bare WSe_2 (black curve) and $WSe_2/[Mo_3S_{13}]^{2-}$ cluster catalyst (red curve) photoelectrodes under chopped illumination (Ar-ion laser, 457.9 nm, 216 mW cm^{-2}). 14

Figure S15. Contact potential difference (CPD) measurement of the $[Mo_3S_{13}]^{2-}$ cluster on FTO measured by Kelvin Probe. 15

Figure S16. Contact potential difference (CPD) of the bare WSe_2 electrode 16

Figure S17. Crystal structure of (A) WSe_2; (B) and (C) Mo_3S_{13} cluster side and top view where up S stands for terminal sulfur facing up and down S stands for bridging sulfur facing down. 17

Figure S18. Six possible adsorption sites of Mo_3S_{13} cluster on WSe_2 monolayer: (A) Down ring; (B) Down W; (C) Down Se; (D) Up ring; (E) Up W; (F) Up Se. The green, gray, yellow, and purple balls represent the Se, W, S, and Mo atoms, respectively. 17

Figure S19. Linear sweep voltammetry (LSV) of bare $[Mo_3S_{13}]^{2-}$ (dark blue curve) and bare WSe_2 (blue curve) and $WSe_2/[Mo_3S_{13}]^{2-}$ photoelectrodes (120 nm, green curve) under front-side illumination conditions using chopped light (AM 1.5) and a 0.5 M H_2SO_4 (pH=0.3) aqueous electrolyte. 18

Table S1. The *Gibbs free* energy ((ΔG^0_{H*} eV) of first H* on *terminal* and *bridging sulfur atoms* of the Mo_3S_{13} cluster. 19

Table S2. The adsorption energy of the possible adsorption sites in Figure S18 (in eV). 19

Table S3. The Bader charge of the Mo_3S_{13} cluster before and after the adsorption on WSe_2 in Figure 6(A). 19

References 19

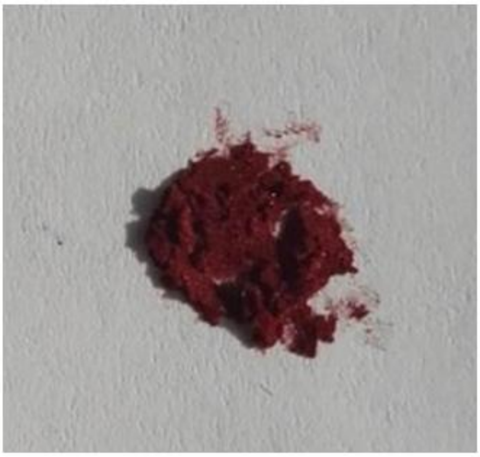

Figure S1. Photograph of synthesized dark red powder of $(NH_4)_2Mo_3S_{13}$

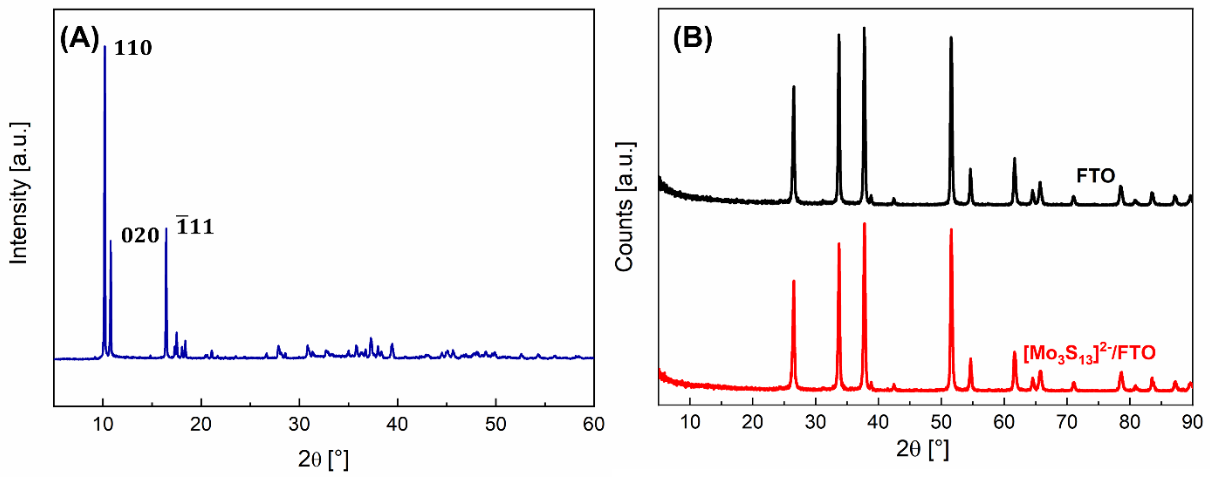

Figure S2. XRD patterns of (A) $(NH_4)_2Mo_3S_{13}$ precursor powder; (B) bare FTO substrate (black); $[Mo_3S_{13}]^{2-}$ cluster film spin-coated on FTO substrate (red).

XRD experimental:

A Bruker AXS D8 Advance X-ray diffractometer with Cu Kα radiation (λ = 0.15406 nm) was used to obtain X-ray diffractograms (XRD). The thin film analysis was measured under grazing incidence configuration with an incidence angle of 0.5°. The detection angle (2θ) was varied from 5° to 90°, while powder samples were measured under θ-2θ coupling from 5° to 60°.

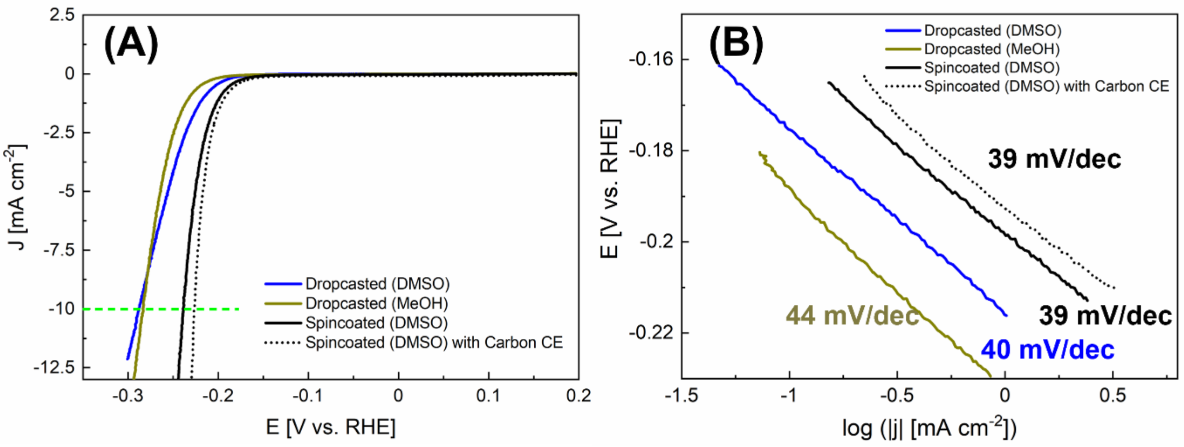

Figure S3. (A) iR-corrected cyclic voltammograms (CV); (B) Tafel plots derived from the CV curves of $[Mo_3S_{13}]^{2-}$ cluster electrodes prepared using DMSO and MeOH as solvents via drop-casting and spin-coating.

In principle, hydrogen evolution is a two-step process.

The first setup featuring a proton reduction (also known as Volmer step):

$$H^+ + e^- + * \rightarrow H^* \quad \text{(S1)}$$

associated with a Tafel slope b = 2.3RT/αF ≈ 120 mV.

The second is the hydrogen desorption step with two alternatives proposed: either by the Heyrovsky step:

$$H^+ + e^- + H^* \rightarrow H_2 \quad \text{(S2)}$$

with b = 2.3RT/(1+α)F ≈ 40 mV,

or the Tafel step formulated as:

$$2H^* \rightarrow H_2 + 2* \quad \text{(S3)}$$

with b= 2.3RT/2F ≈ 30 mV,

where the asterisks * in these equations stand for active sites on the catalyst surface, R is the ideal gas constant (8.314 J K^{-1} mol^{-1}), T is the temperature in Kelvin, and α is the barrier symmetry factor (~0.5 for metals).[1]

The sample prepared by drop-casting using methanol (MeOH) as solvent has a slightly higher Tafel slope value (44 mV/dec). Possible reason for this could be that the lower solubility of the $(NH_4)_2Mo_3S_{13}$ precursor in MeOH and the fast evaporation of the solvent during the drop-casting process which lead to inhomogeneity in the deposited film.

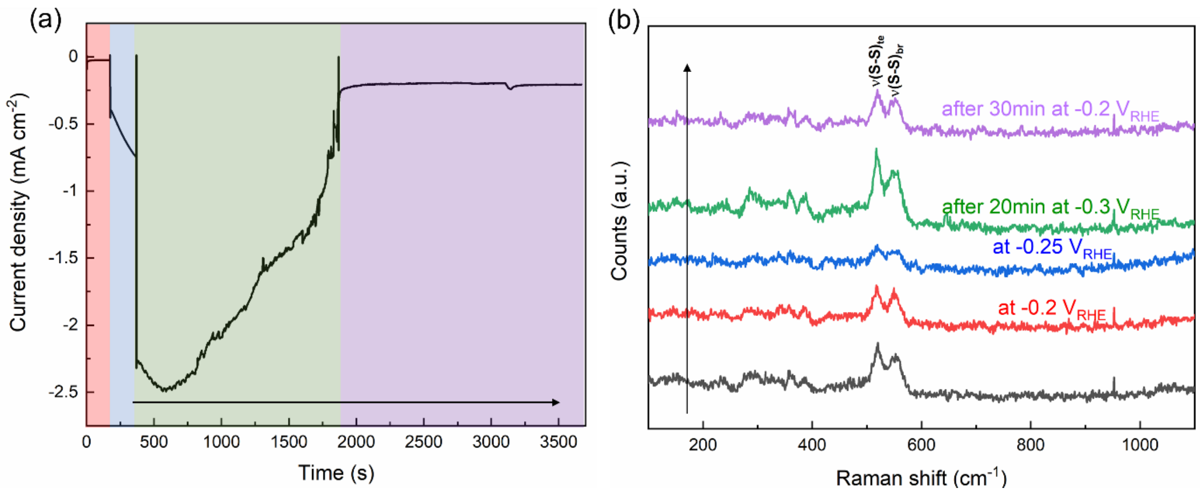

Figure S4. (a) Current density of $[Mo_3S_{13}]^{2-}$ cluster catalyst on FTO measured in Raman cell under various potential: -0.2 V vs. RHE (red), -0.25 V vs. RHE (blue), -0.3 V vs. RHE (green), -0.2 V vs. RHE (purple); (b) corresponding in-situ Raman spectra measured in the cell with electrolyte (pH 0.3, 0.5 M H_2SO_4)

In-situ Raman:

Figure S4b, the black curve was measured before the potentiostatic tests while the red and blue curves are the Raman spectra measured at -0.2 V and -0.25 V vs. RHE respectively. After that, the sample was kept at -0.3 V vs. RHE for around 25 min. Due to the fast bubble formation in a thin chamber with very small amount of electrolyte, the current dropped dramatically, and the horizontal chamber was almost filled up with gas even with a constant electrolyte pumping. Therefore after 25 min, the electrochemical test was stopped manually to prevent the electrode from damage. The green curve of the Raman spectra was taken afterwards. Finally in the last step, we kept the electrode at a moderate potential (-0.2 V vs. RHE) for 30 min so that the gas bubbles won't block the whole chamber and then measured the Raman spectra (purple). As shown by all the spectra in Figure S4b, although the blue and purple spectra show slightly lower signals, possibly due to light scattering from bubble formation or photo-corrosion after long term test under Raman laser, both terminal and bridging $[S_2]^{2-}$ modes exist no matter during potentiostatic measurements or after long term measurements, which indicates a good stability of the cluster catalyst and proves that the terminal $[S_2]^{2-}$ mode is indeed the active center in the case of $[Mo_3S_{13}]^{2-}$ cluster, unlike the amorphous MoS_x which almost completely lost its cluster-like structure after electrochemical cycling.

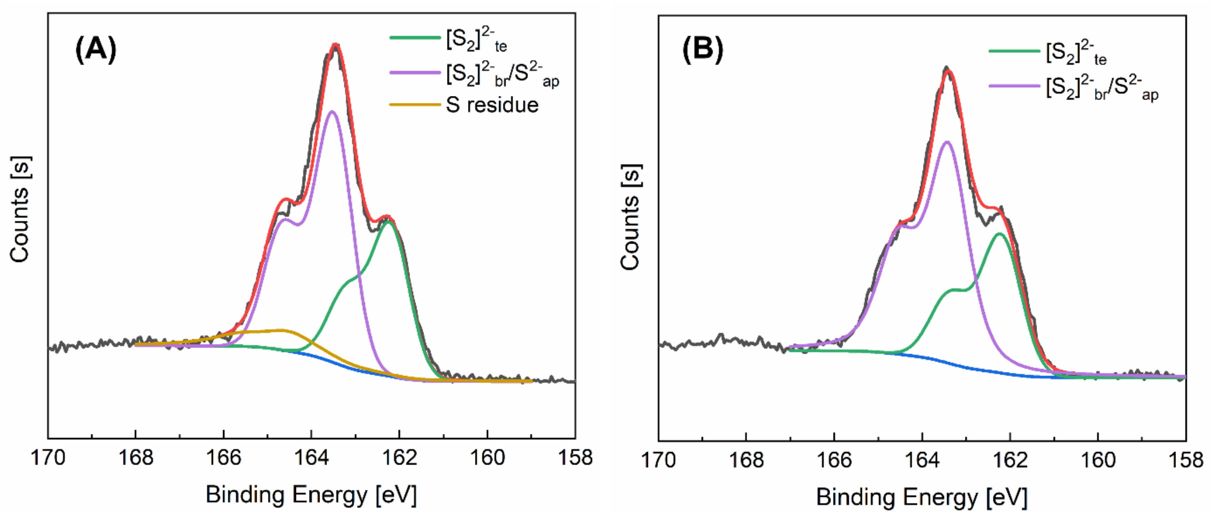

Figure S5. XPS S2p spectra of the $[Mo_3S_{13}]^{2-}$ cluster catalyst on FTO: (A) electrode as-prepared; (B) after 10 CV cycles. Terminal $[S_2]^{2-}$ units are represented by the green curve while bridging $[S_2]^{2-}$ and apical S^{2-} entities are shown by purple curve in the deconvoluted XPS spectra. Residual sulfur is shown by the dark yellow curve in Figure (A).

XPS experimental:

X-ray photoelectron spectroscopy (XPS) was carried out with a monochromatic Al Kα X-ray source (1486.74 eV, Specs Focus 500 monochromator). A hemispherical analyser (Specs Phoibos 100) in an ultrahigh vacuum system (base pressure of about 10^{-8} mbar) was used to study the composition and the valence states of different elements.

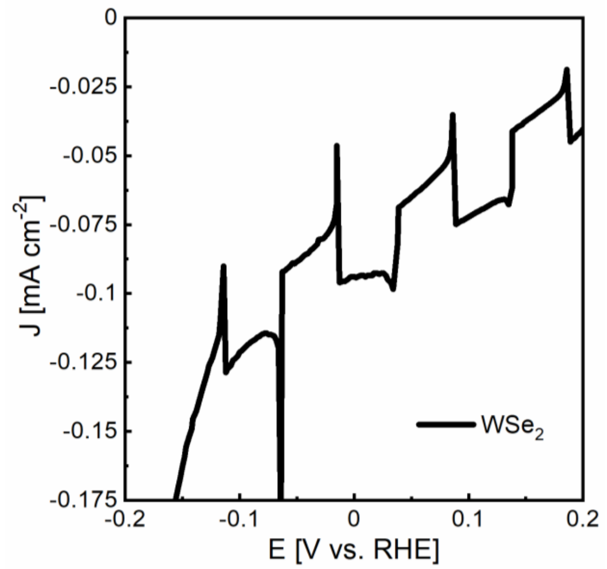

Figure S6. Magnified LSV of a bare WSe_2 photocathode on TiO:N/Si substrate under front side AM1.5 chopped illumination in 0.5 M H_2SO_4 aqueous electrolyte (pH 0.3).

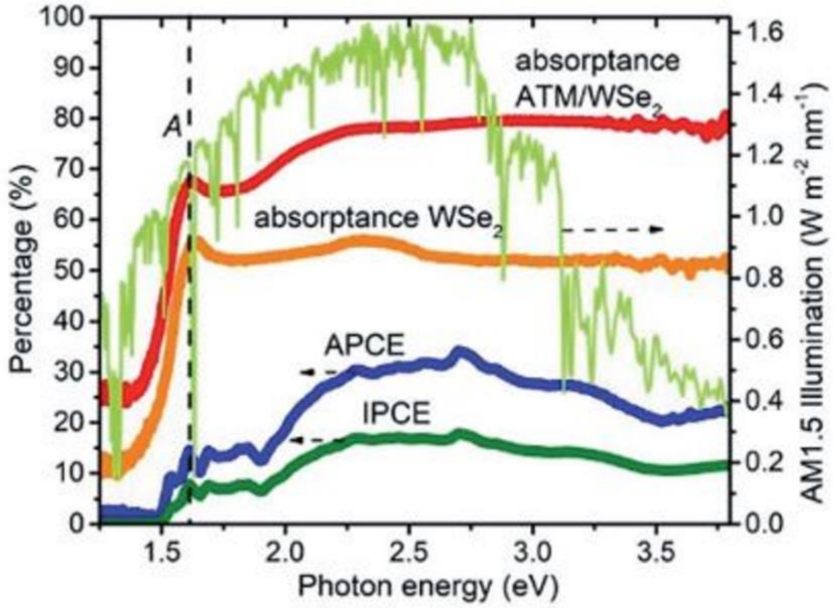

Figure S7. Absorptance spectra, IPCE and APCE spectra of WSe_2, $WSe_2/[Mo_3S_{13}]^{2-}$ photocathodes.[2]

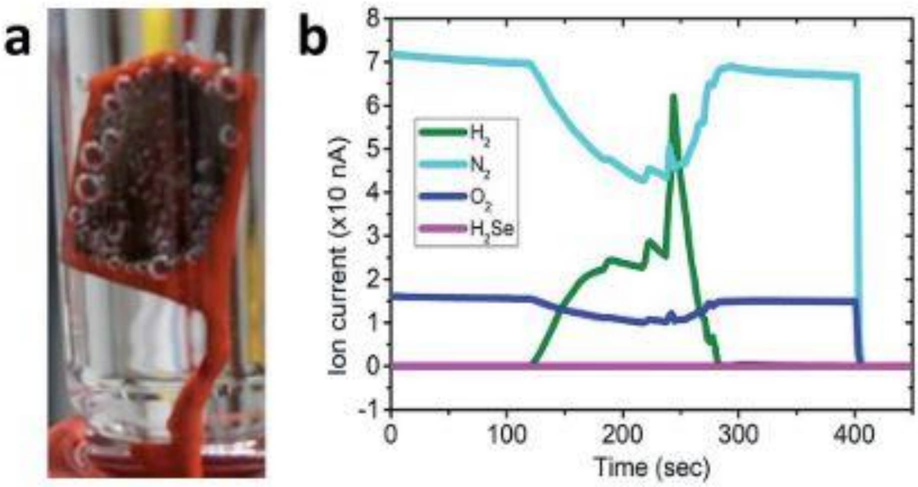

Figure S8. (a) Gas formation at the electrode surface; (b) mass signal of the gas products vs. time (s).[2]

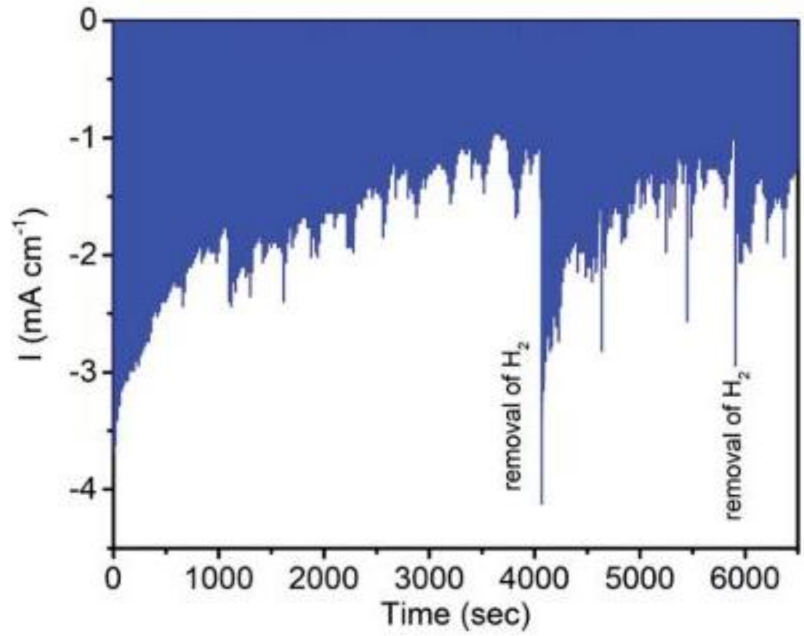

Figure S9. Stability test of a WSe_2 (200 nm)/$[Mo_3S_{13}]^{2-}$ photocathode at 0 V vs. RHE in 0.5 M H_2SO_4 under AM 1.5 illumination.[2]

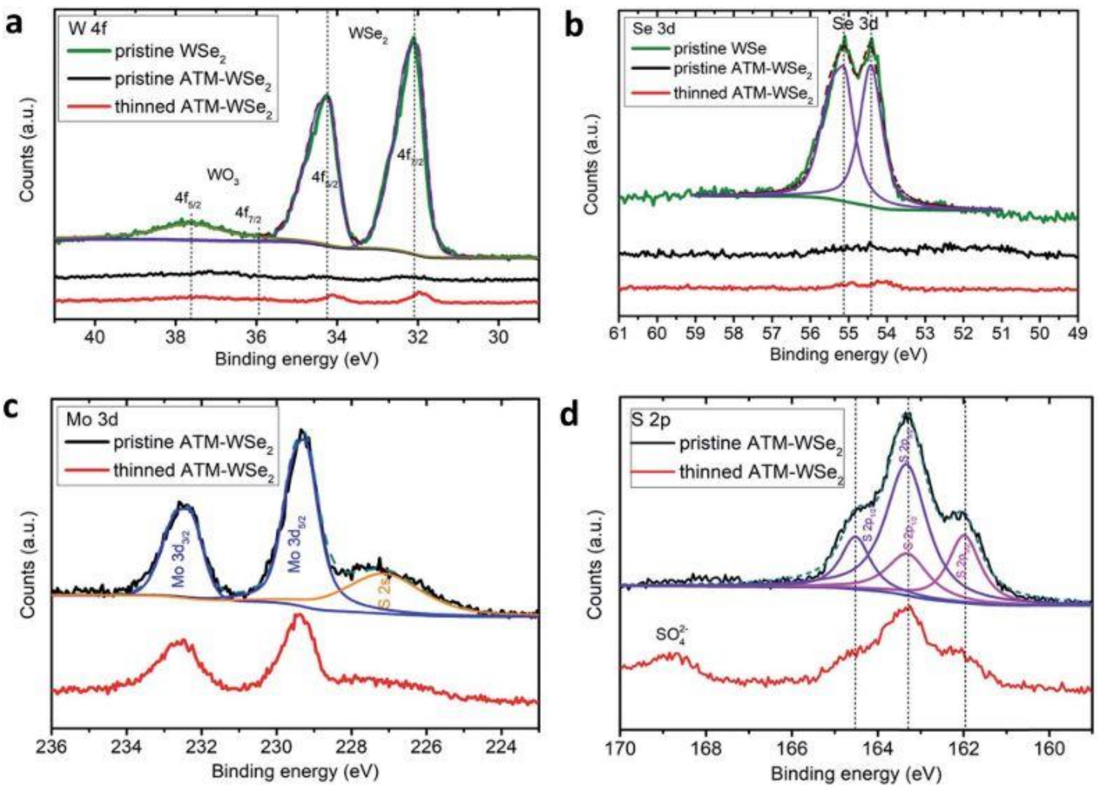

Figure S10. X-ray photon spectra (XPS) of WSe_2, $WSe_2/[Mo_3S_{13}]^{2-}$ before and after PEC: (a) W 4f, (b) Se 3d, (c) Mo 3d, and (d) S 2p.[2] Thinned ATM-WSe_2 stands for $WSe_2/[Mo_3S_{13}]^{2-}$ photocathode after PEC.

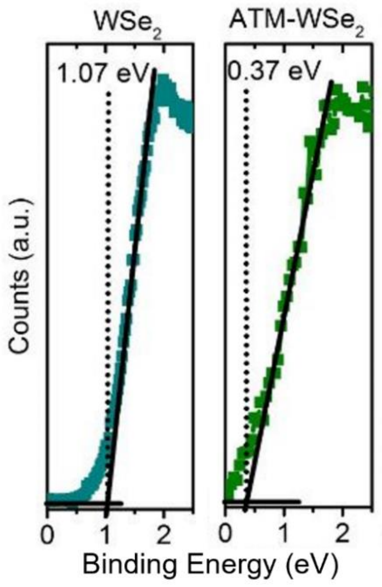

Figure S11. Ultraviolet photoelectron spectroscopy (UPS) of WSe_2 and $WSe_2/[Mo_3S_{13}]^{2-}$ to determine the valence band edge position.[2]

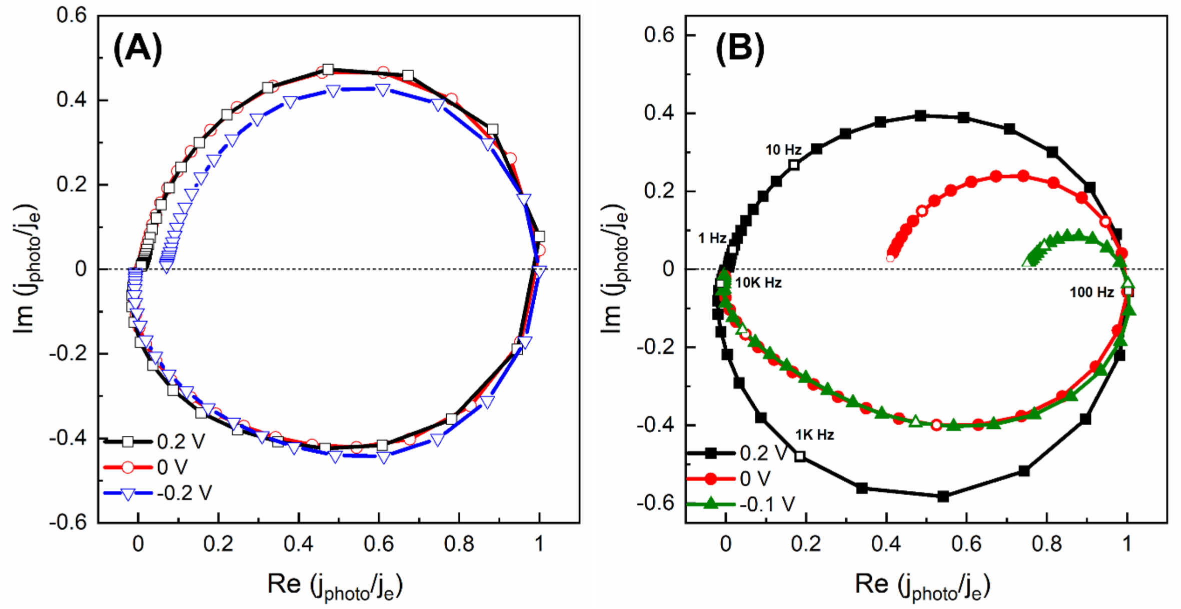

Figure S12. IMPS spectra of (A) a bare WSe_2 and (B) of a $WSe_2/[Mo_3S_{13}]^{2-}$ photocathode under modulated illumination at different potentials vs. RHE.

In IMPS measurements, the illumination intensity is modulated by an amplitude of 10%. The photocurrent at a fixed potential was recorded as a function of frequency which varies from 100 kHz to 1 Hz. The overall modulated photocurrent consists of both the minority charge and the majority charge current, where the latter normally precedes the minority current. Therefore, under fluctuated illumination, the resulted photocurrent could be expressed as a real and an imaginary part. In a typical IMPS spectrum, the imaginary photocurrent is plotted as a function of the real part of the photocurrent. The IMPS spectra of WSe_2 and $WSe_2/[Mo_3S_{13}]^{2-}$ electrodes, measured at different applied potentials, are shown in Figure S12A and S12B. In these two figures, the intercepts of high frequency semi-circles are normalized to 1 for easier comparison between spectra measured at different applied potentials by dividing both the real and imaginary photocurrent with the electron current. The electron current can be demonstrated by the absolute intercepts of the high frequency semicircles with the x-axis, which corresponds to the flux of electrons that arrive at the semiconductor interface before they recombine or be transferred to the electrolyte (see Figure S13).[3] The low frequency upper semi-circle (note that the sign of the y-axis is inverted) is dominant by recombination processes. The intercept of this semi-circle stands for k_{tr} / (k_{tr} + k_{rec}) which equals to η_{CT} according to Eq. 1. Besides, the imaginary photocurrent reaches its maximum when the frequency matches the characteristic relaxation constant of the system as depicted by Eq. S4 (see also Figure S13).

$$\omega_{max} = k_{tr} + k_{rec} \quad \text{(S4)}$$

In Figure S12A, it is shown that the recombination semi-circles varying the potentials applied for the bare WSe_2 electrode almost have the same shape and size and the intercepts of the recombination semi-circles are close to zero. This behavior suggests that the performance of the bare WSe_2 photocurrent is largely suppressed by surface recombination. The high recombination rate could be caused by palladium in the layer which was used as promoter for WSe_2 crystallization. Pd forms a $PdSe_x$ phase due to the reaction with H_2Se, which is a component of the reacting gas in the sputtering system. The $PdSe_x$ phase mainly accumulated on the WSe_2 surface which could act as recombination centers.[4] From Figure S12B, it can clearly be seen that the the upper semicircles indicate a lower recombination proportion compared to bare WSe_2 in Figure S12A, indicating that the $[Mo_3S_{13}]^{2-}$ cluster catalyst inhibits the recombination process on the surface of WSe_2.

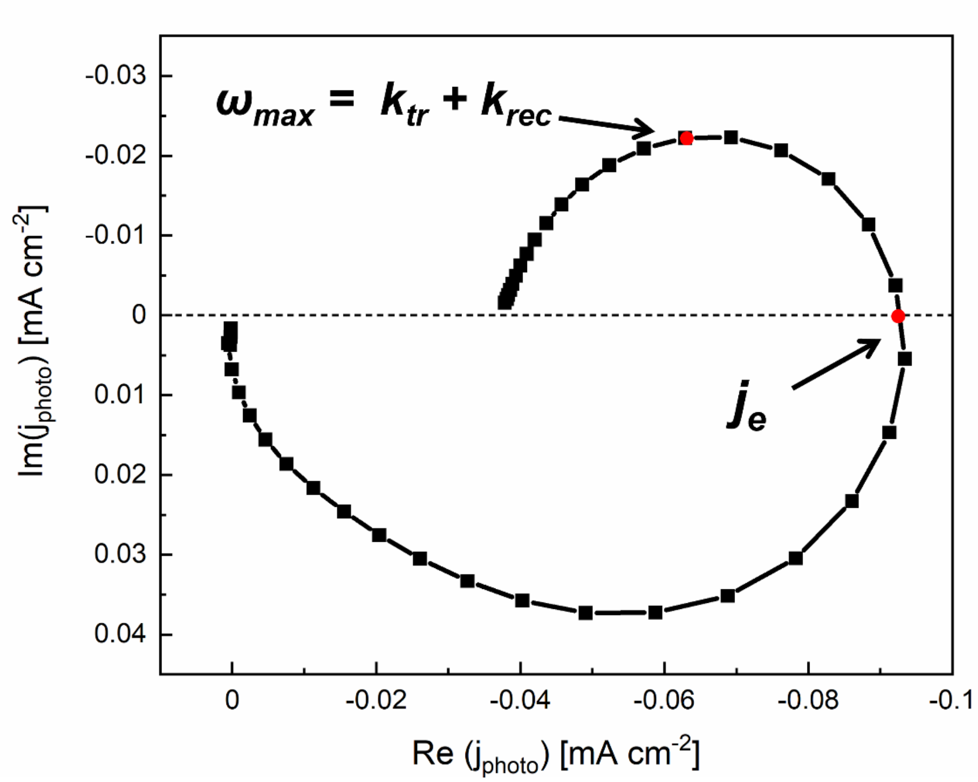

Figure S13. An example of a raw IMPS spectrum of the $WSe_2/[Mo_3S_{13}]^{2-}$ electrode indicating the values of the electron current (j_e) and of ω_{max} by the red dots (also see Eq. 1 and Eq. S4).

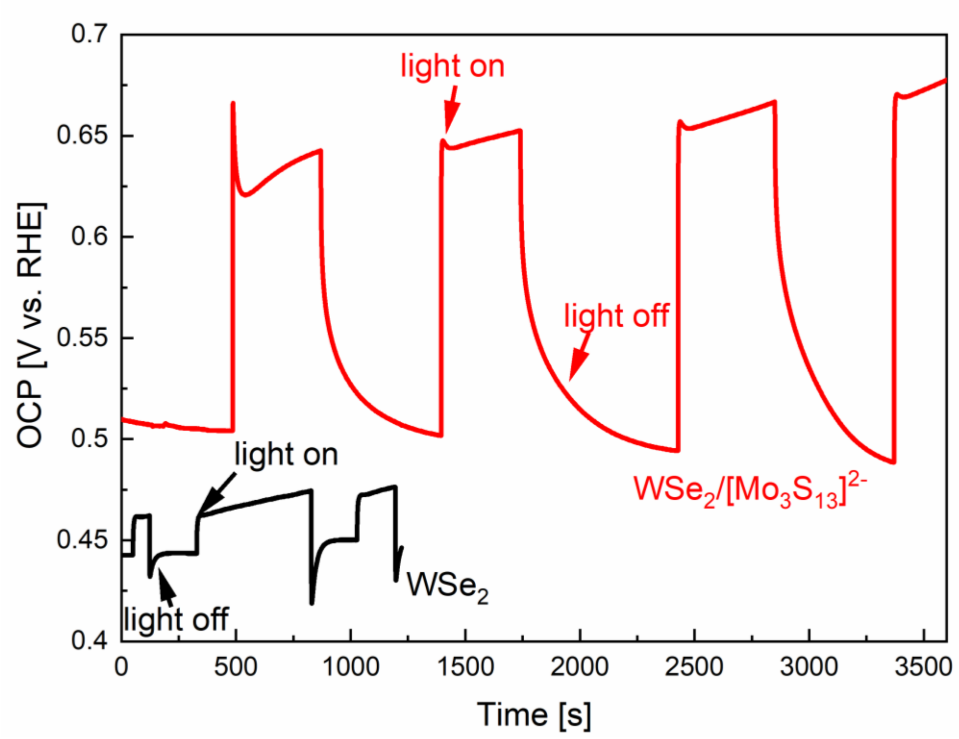

Figure S14. The open circuit potential difference (ΔOCP) measurements of bare WSe_2 (black curve) and $WSe_2/[Mo_3S_{13}]^{2-}$ cluster catalyst (red curve) photoelectrodes under chopped illumination (Ar-ion laser, 457.9 nm, 216 mW cm^{-2}).

The ΔOCP values can be determined by the OCP differences of the sample between light on and off mode. For instance, the OCP of WSe_2 sample under dark condition is around 0.44 V vs. RHE while its OCP under illumination is around 0.46 V vs. RHE which means that the ΔOCP of WSe_2 is around 20 mV. The ΔOCP of $WSe_2/[Mo_3S_{13}]^{2-}$ is calculated to be around 150 mV.

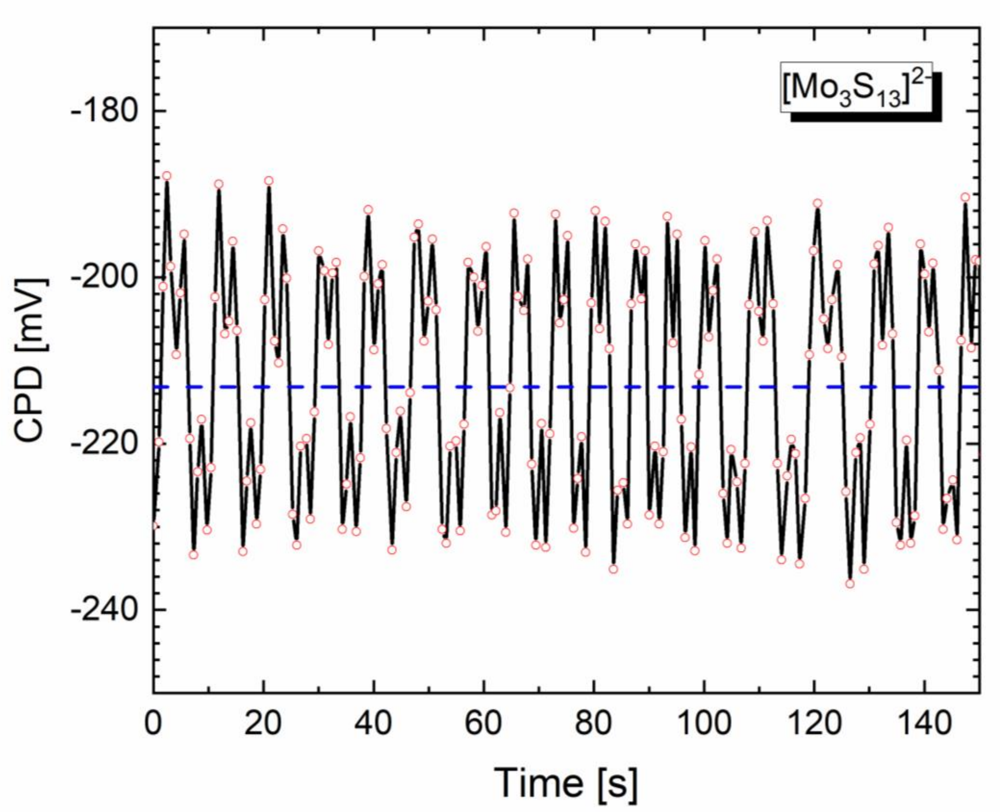

Figure S15. Contact potential difference (CPD) measurement of the $[Mo_3S_{13}]^{2-}$ cluster on FTO measured by Kelvin Probe.

The work function of the $[Mo_3S_{13}]^{2-}$ cluster film was determined by Eq. S5 expressed as below:

$$\Phi_{sample} = \Phi_{tip} + e \cdot CPD \tag{S5}$$

The CPD value could be determined by the mean value in Figure S15, which is around -213 ± 14 mV. The work function of the Kelvin probe measured amounted to 4.90 ± 0.03 eV. Accordingly, the work function of the thin $[Mo_3S_{13}]^{2-}$ film is 4.69 ± 0.03 eV.

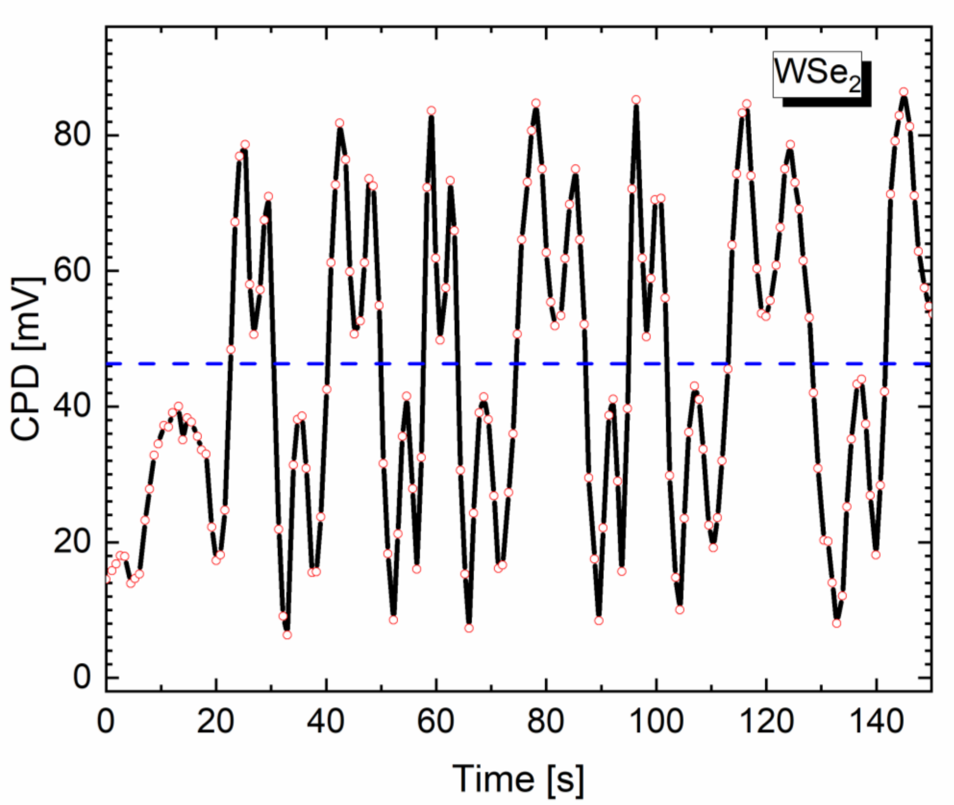

Figure S16. Contact potential difference (CPD) of the bare WSe_2 electrode

Measured $\Phi_{tip} = 5.03 \pm 0.03$ eV; Measured CPD on WSe_2 =47±22 mV.
Therefore, the work function of WSe_2 could be determined from Figure S16 and Eq. S5, which is 5.08 ± 0.04 eV. The VBM of WSe_2 is 5.34 ± 0.03 eV which is 0.26 ± 0.05 eV below the Fermi level.

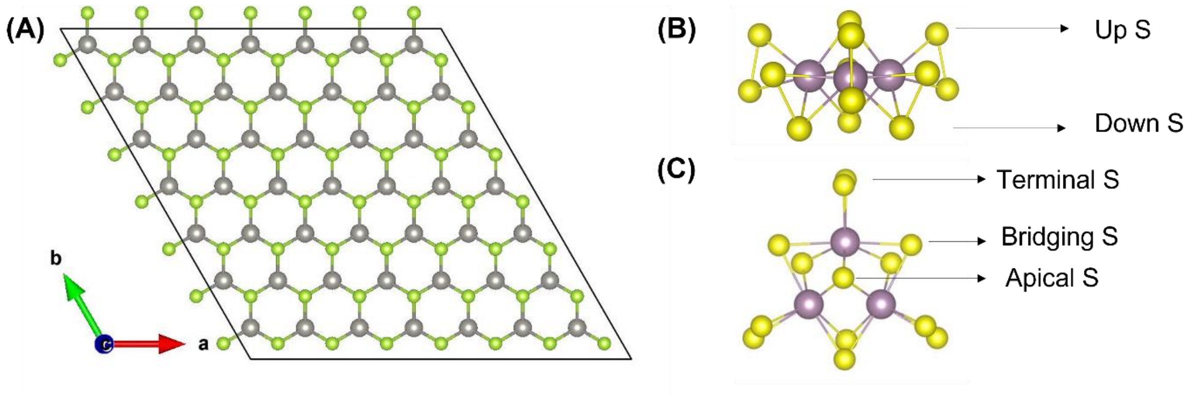

Figure S17. Crystal structure of (A) WSe_2; (B) and (C) Mo_3S_{13} cluster side and top view where up S stands for terminal sulfur facing up and down S stands for bridging sulfur facing down.

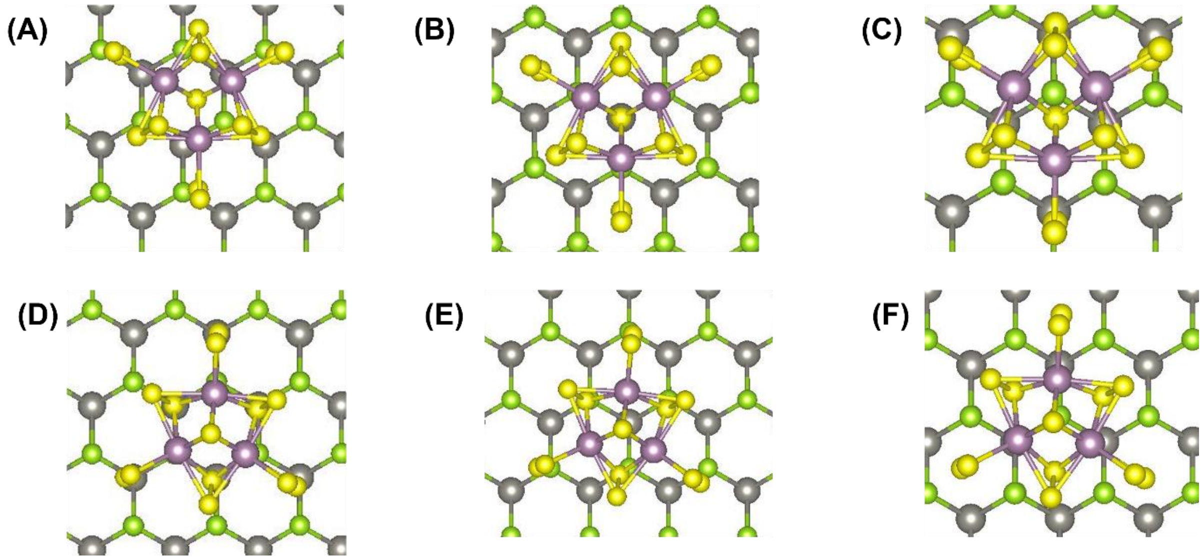

Figure S18. Six possible adsorption sites of Mo_3S_{13} cluster on WSe_2 monolayer: (A) Down ring; (B) Down W; (C) Down Se; (D) Up ring; (E) Up W; (F) Up Se. The green, gray, yellow, and purple balls represent the Se, W, S, and Mo atoms, respectively.

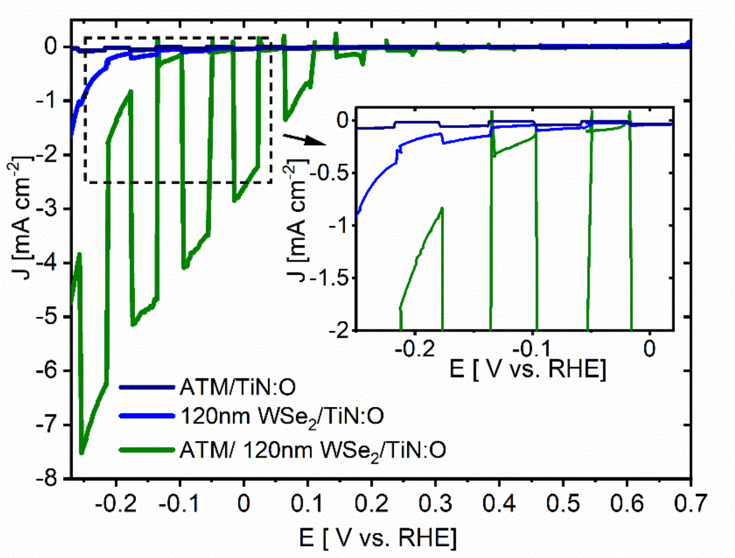

Figure S19. Linear sweep voltammetry (LSV) of bare $[Mo_3S_{13}]^{2-}$ (dark blue curve) and bare WSe_2 (blue curve) and $WSe_2/[Mo_3S_{13}]^{2-}$ photoelectrodes (120 nm, green curve) under front-side illumination conditions using chopped light (AM 1.5) and a 0.5 M H_2SO_4 (pH=0.3) aqueous electrolyte.

Table S1. The *Gibbs free* energy (ΔG^0_{H*} eV) of first H* on *terminal* and *bridging sulfur atoms* of the Mo_3S_{13} cluster.

	ΔG^0_{H*} [eV]	
Sulfur positions of the cluster	Mo_3S_{13}	Mo_3S_{13}@WSe_2
**t_up**	-0.117	-0.102
**b_up**	1.92	0.885

Further details of simulation on the single Mo_3S_{13} cluster are described in our previous publication[5]: "t" stands for terminal sulfur, and "b" for bridging sulfur of S_2^{2-} units from the Mo_3S_{13} cluster (shown in Figure S17(B, C)).

Table S2. The adsorption energy of the possible adsorption sites in Figure S18 (in eV).

	Down ring (A)	Down W (B)	Down Se (C)	Up ring (D)	Up W (E)	Up Se (F)
**E_{ad} (eV)**	**-0.73**	**-0.83**	**-0.82**	**-0.88**	**-0.62**	**-0.89**

Table S3. The Bader charge of the Mo_3S_{13} cluster before and after the adsorption on WSe_2 in Figure 6(A).

	**Before adsorption**	**After adsorption**
**Mo**	4.927	4.927
**Apical S**	6.566	6.572
**up bridging S**	6.216	6.227
**up terminal S**	6.194	6.207
**down bridging S**	6.236	6.237
**down terminal S**	6.239	6.239

References

[1] D. Merki, H. Vrubel, L. Rovelli, S. Fierro, X. Hu, *Chem. Sci.* **2012**, *3*, 2515.

[2] F. Bozheyev, F. Xi, P. Plate, T. Dittrich, S. Fiechter, K. Ellmer, *J. Mater. Chem. A* **2019**, *7*, 10769.

[3] H. K. Dunn, J. M. Feckl, A. Müller, D. Fattakhova-Rohlfing, S. G. Morehead, J. Roos, L. M. Peter, C. Scheu, T. Bein, *Phys. Chem. Chem. Phys.* **2014**, *16*, 24610.

[4] F. Bozheyev, D. Friedrich, M. Nie, M. Rengachari, K. Ellmer, *Phys. Status Solidi Appl. Mater. Sci.* **2014**, *211*, 2013.

[5] F. Xi, P. Bogdanoff, K. Harbauer, P. Plate, C. Höhn, J. Rappich, B. Wang, X. Han, R. Van De Krol, S. Fiechter, *ACS Catal.* **2019**, *9*, 2368.
